# A taxonomy has been developed for outcomes in medical research to help improve knowledge discovery

**DOI:** 10.1016/j.jclinepi.2017.12.020

**Published:** 2018-04

**Authors:** Susanna Dodd, Mike Clarke, Lorne Becker, Chris Mavergames, Rebecca Fish, Paula R. Williamson

**Affiliations:** a MRC North West Hub for Trials Methodology Research, Department of Biostatistics, Institute of Translational Medicine, University of Liverpool, Liverpool L69 3GS, UK; b School of Medicine, Dentistry and Biomedical Sciences, Centre for Public Health Institute for Health Sciences, Northern Ireland Methodology Hub, Queen's University Belfast, Belfast, UK; c Department of Family Medicine, SUNY Upstate Medical University, Syracuse, NY, USA; d Department of Informatics and Knowledge Management, Cochrane Central Executive, Freiburg, Germany; e Division of Molecular and Clinical Cancer Sciences, University of Manchester, Manchester, UK

**Keywords:** Randomized controlled trials, Outcomes, Effectiveness trials, PICO, Taxonomy, COMET, Cochrane, Core outcome sets, Systematic reviews, Classification, Comparative effectiveness research

## Abstract

**Objectives:**

There is increasing recognition that insufficient attention has been paid to the choice of outcomes measured in clinical trials. The lack of a standardized outcome classification system results in inconsistencies due to ambiguity and variation in how outcomes are described across different studies. Being able to classify by outcome would increase efficiency in searching sources such as clinical trial registries, patient registries, the Cochrane Database of Systematic Reviews, and the Core Outcome Measures in Effectiveness Trials (COMET) database of core outcome sets (COS), thus aiding knowledge discovery.

**Study Design and Setting:**

A literature review was carried out to determine existing outcome classification systems, none of which were sufficiently comprehensive or granular for classification of all potential outcomes from clinical trials. A new taxonomy for outcome classification was developed, and as proof of principle, outcomes extracted from all published COS in the COMET database, selected Cochrane reviews, and clinical trial registry entries were classified using this new system.

**Results:**

Application of this new taxonomy to COS in the COMET database revealed that 274/299 (92%) COS include at least one physiological outcome, whereas only 177 (59%) include at least one measure of impact (global quality of life or some measure of functioning) and only 105 (35%) made reference to adverse events.

**Conclusions:**

This outcome taxonomy will be used to annotate outcomes included in COS within the COMET database and is currently being piloted for use in Cochrane Reviews within the Cochrane Linked Data Project. Wider implementation of this standard taxonomy in trial and systematic review databases and registries will further promote efficient searching, reporting, and classification of trial outcomes.

**What's new?**

**Key findings**
• Existing taxonomy structures are intended as general health research vocabularies, rather than focusing on outcomes; do not provide sufficiently granular or comprehensive classification of trial outcomes; or are disease specific or focused on patient-centered outcomes only.
• The current lack of an outcome classification system, fit for purpose, is holding back research as a result of (i) inconsistency and ambiguity in how outcomes are described across different studies and (ii) inefficiency in searching knowledge sources including the published literature and ongoing research repositories such as clinical trials registries, which to date include outcomes as free-text entries only.
• A new workable outcome taxonomy is proposed, the robustness of which has been demonstrated through application to a large number of trial registry entries in clinicaltrials.gov, Cochrane Reviews, and core outcome sets in the Core Outcome Measures in Effectiveness Trials (COMET) database.

**What this adds to what was known?**
• Core outcome set developers should give more attention to measures of life impact and adverse events when determining core outcome sets for trials of the effectiveness of health and social care interventions.

**What is the implication and what should change now?**
• An accepted taxonomy of outcomes would increase the reuse value of outcome data, just as Medical Subject Headings terms have transformed the searchability of medical literature. Wider implementation of this taxonomy will help to reduce waste in research by promoting efficient searching, reporting, and classification of clinical outcomes for the first time, thereby speeding up research activities including discovery science and “big data” approaches to extracting knowledge from published information.

## 1 Background

Recognition that insufficient attention has been paid to the choice of outcomes to measure in clinical trials is increasing. In the context of clinical trials, an outcome is defined to be a measurement or observation used to capture and assess the effect of treatment such as assessment of side effects (risk) or effectiveness (benefits) [1]. The Core Outcome Measures in Effectiveness Trials (COMET) Initiative [E1], [2] brings together people interested in the development and application of agreed standardized sets of outcomes, known as “core outcome sets” (COS). These sets represent the minimum that should be measured and reported in all clinical trials of a specific condition and are also suitable for use in clinical audit or research other than randomized trials. One of the successes of COMET has been the development of a publicly available searchable database of completed and ongoing projects in COS development [2], [3], [4], [5], [6], [7]. This unique resource provides information on the COS developed to date and is currently searchable by population, intervention, and condition. However, as yet the records in the COMET database have not been categorized according to outcome, the fourth of the essential elements that should be defined for a trial, according to the population, intervention, comparison, outcome (PICO) model.

Similarly, outcomes in trial registries (including the EU Clinical Trials Register, ClinicalTrials.gov and ISRCTN registry) can be entered as free text only, hampering the ability to search for outcomes effectively because of variation and inconsistencies in how outcomes are described across different trials. Over 60% queries related to requests to register a trial relate to how outcomes were described [8]. Standardized terminology to describe outcomes is starting to come into use in preclinical research, where variations in description have impeded computational analysis of phenotypic data [9]. However, there is currently no consensus on how clinical trial outcomes should be classified. Standard terminology to describe outcomes in preclinical and clinical research would facilitate the comparison of outcomes between preclinical and clinical settings, potentially providing insight into the reasons why so many late-phase trials “fail” despite promising results from preclinical studies.

A taxonomy is a scheme of classification that is often used for, for example, the naming of living organisms but which can also be used as a controlled vocabulary (i.e., an authoritative list of terms for use in indexing) with a hierarchical structure [E2]. Taxonomies exist for many aspects of health research, such as the International Classification of Functioning, Disability and Health, (ICF [E3]) and International Statistical Classification of Diseases and Related Health Problems 10th Revision (ICD-10, [E4]). The Cochrane Linked Data Project [E5] uses a 15-item taxonomy for high-level categorization of interventions (for the IC components of PICO).

A standard outcome taxonomy would help to improve knowledge discovery by facilitating organized searching of trials by outcome in trial registries and databases. For example, a researcher might be interested in identifying all interventions that have been tested in a randomized trial to improve a particular outcome. Similarly, COS have the potential to reduce research waste by avoiding unnecessary duplication of efforts and allowing the results of clinical trials to be combined [2], [7], but this benefit will only be realized with COS uptake. Classification of trial outcomes will facilitate efficient assessment of COS uptake, again improving knowledge [10].

We sought to identify and further develop as necessary a taxonomy providing sufficient granularity and scope for the classification of all outcomes in the COS listed in the COMET database, which would be equally suitable for classification of outcomes included in trial registries, trial reports, and systematic reviews. This taxonomy is intended for the classification of what, rather than how, outcomes are measured.

## 2 Methods

A suitable taxonomy for clinical trial outcomes must clearly differentiate between high-level outcome types, while comprehensively covering all potential outcomes from clinical trials in a sensible hierarchical structure. We carried out a literature review to identify existing outcome taxonomies that would inform the development of the one presented here. We searched PubMed for published journal articles and Internet resources such as Google. Our search involved a combination of terms, including “ontology”, “taxonomy”, “classification” or “categorisation” and “health”, “health research”, “trial”, and “outcomes”.

To examine outcome classification systems used as part of COS development, the COS studies within the COMET database that included a systematic or literature review to identify relevant studies were reviewed to determine how they categorized their outcomes.

The lack of an existing suitable outcome taxonomy for trial outcomes led to the subsequent development of a new taxonomy to classify trial and systematic review outcomes. This was an iterative process, starting with the 15-category scale developed by Smith et al. [11] to classify outcomes recorded in Cochrane Reviews (Table 1). A refined version of this scale, developed by two authors (P.R.W. and M.C.), was piloted as part of PICO classification of reviews within the Cochrane database [E5]. This 12-category version was then further developed to provide more detail relating to physiological, function, and resource use domains, leading to a taxonomy with 38 outcome domains within five core areas. Explanations and examples of outcomes within each of these domains are found in Supplementary Table 1 on the journal's web site at www.elsevier.com.

**Table 1:** Development of 38-category scale

Core area	Smith	Williamson/Clarke (initial)	Williamson/Clarke (revised)
Death	1: Mortality/survival	1: Mortality/survival	1: Mortality/survival
Physiological or clinical	2: Physiological/clinical	2: Physiological/clinical	2–24: **Physiological/clinical** 2: Blood and lymphatic system outcomes 3: Cardiac outcomes 4: Congenital, familial and genetic outcomes 5: Endocrine outcomes 6: Ear and labyrinth outcomes 7: Eye outcomes 8: Gastrointestinal outcomes 9: General outcomes 10: Hepatobiliary outcomes 11: Immune system outcomes 12: Infection and infestation outcomes 13: Injury and poisoning outcomes 14: Metabolism and nutrition outcomes 15: Musculoskeletal and connective tissue outcomes 16: Outcomes relating to neoplasms: benign, malignant and unspecified (including cysts and polyps) 17: Nervous system outcomes 18: Pregnancy, puerperium, and perinatal outcomes 19: Renal and urinary outcomes 20: Reproductive system and breast outcomes 21: Psychiatric outcomes 22: Respiratory, thoracic and mediastinal outcomes 23: Skin and subcutaneous tissue outcomes 24: Vascular outcomes
	3: Infection	3: Infection	
	4: Pain	4: Pain	
Life impact	5: Activities of daily living	5: Function	**Functioning** 25: Physical functioning 26: Social functioning 27: Role functioning 28: Emotional functioning/well-being 29: Cognitive functioning
		- Physical	
		- Social	
		- Role	
	6: Psychosocial	6: Psychosocial	
		7: Mental health	
	7: QoL	8: HRQL	30: Global quality of life
			31: Perceived health status
	8: Compliance	9: Compliance (including withdrawal from treatment)	32: Delivery of care, including - Satisfaction/patient preference - Acceptability and availability - Adherence/compliance - Withdrawal from treatment - Appropriateness of treatment - Process, implementation, and service outcomes
	9: Withdrawal from treatment/study		
	10: Satisfaction (patient, carer, health care provider)	10: Satisfaction	
			33: Personal circumstances
Resource use	11: Medication	11: Resource use - Economic - Hospital - Operative - Medication	**Resource use** 34: Economic 35: Hospital 36: Need for further intervention 37: Societal/carer burden
	12: Economic		
	13: Hospital		
	14: Operative		
Adverse events	15: Adverse events/effects	12: Adverse events/effects	38: Adverse events/effects

Physiological outcomes are categorized according to the underlying cause or affected body system, grouped using the MedDRA System Organ Classes (SOCs) (Supplementary Table 2 on the journal's web site at www.elsevier.com) with the exception of four SOCs (Investigations, Social circumstances, Surgical and medical procedures, Product issues) which are not considered relevant within the physiological/clinical domains. For example, “endocrine outcomes” are those associated with endocrine disorders. “Outcomes related to neoplasms” include those relating to physiological function, signs and symptoms caused by benign, malignant and unspecified (including cysts and polyps) neoplasms, including solid and nonsolid tumors. Examples of such outcomes include “time to recurrence”, “response rate”, and “clearance of resection margins”. “General outcomes” include those affecting the whole body which cannot be attributed to a certain body system, for example, fatigue, chills, flu-like symptoms, malaise, anorexia, pain (unspecified, not associated with a particular body system), fever (not attributable to infection), anthropometric measures (e.g., weight), “global” measures, “symptoms” (not associated with a particular body system), “physical health”, and fitness. Laboratory parameters (e.g., from blood samples) and scientific measures (e.g., pharmacokinetic outcomes) should be classified within the physiological domain that captures the reason for the assessment (rather than within the “blood and lymphatic system” category, for example).

The functioning categories were extended beyond those used by Smith et al. [11] (activities of daily living and psychosocial) to differentiate more accurately between physical, social, role, emotional, and cognitive functioning.

The “delivery of care” domain contains a number of variables related to health care interventions, including compliance, withdrawal, and satisfaction. These were grouped as they are all related to the appropriateness and acceptability of the intervention and may not be easily distinguishable (e.g., because of overlap between issues relating to compliance, satisfaction with care, withdrawal, treatment failure). Examples of outcomes in this category include patient preference; withdrawal from intervention (e.g., time to treatment failure, reason for stopping therapy); appropriateness, accessibility, quality, and adequacy of intervention; patient or carer satisfaction; and process, implementation, and service outcomes.

The “adverse event” domain includes outcomes broadly labeled as some form of unintended consequence of the intervention (e.g., adverse events/effects, adverse reactions, safety, harm, negative effects, toxicity, complications, sequelae). Specifically named adverse events are classified within the appropriate taxonomy domain relating to the specific event type, with an additional level of categorization which identifies this outcome as an adverse event.

The “mortality/survival” domain includes overall (all-cause) and cause-specific survival/mortality, as well as composite survival outcomes that include death (e.g., disease-free survival). Composite outcomes should be classified in all domains relating to each of the included event types; for example, disease-free survival would be classified within the “mortality/survival” domain as well as the physiological outcome domain relating to the particular disease.

The final 38-item scale was applied to the classification of trial outcomes recorded within the 299 published COS in the COMET database that were published before 2016 and to outcomes from 3,515 Cochrane reviews as part of the pilot phase of the Cochrane Linked Data Project. To further illustrate its applicability, the taxonomy has been applied to outcomes listed in 30 studies identified from a search of the US National Institutes of Health clinical trials registry (www.clinicaltrials.gov). Furthermore, two case studies are presented to demonstrate how the taxonomy can provide standard classification of outcomes across different research settings linked to particular clinical areas. One of the authors (S.D.) assessed all the outcomes in the COS database and National Institutes of Health clinical trial registry. In cases of any doubt or ambiguity, a second opinion (P.R.W.) was sought. Cochrane review outcomes were classified by Cochrane reviewers.

## 3 Results

### 3.1 Literature review

A review of the literature identified several vocabularies (such as MedDRA [E6], and SNOMED CT, [E7]) which exist to organize and classify text relating to health research, many of which are included within the Unified Medical Language System (UMLS, [E8]). However, few relate specifically to outcome classification. For example, the ICF provides a conceptual framework for understanding and describing health and disability, accounting for both patient and contextual factors, rather than an explicit classification of trial outcomes [E3]. Medical Subject Headings (MeSH, the National Library of Medicine's controlled vocabulary thesaurus, [E9]) categories extend beyond health outcomes, covering not only anatomy, diseases, and health care but also technology, occupations, information science, geographicals and so forth. Subclasses within their “diseases” category are similar to our physiological/clinical domains but with additional levels of differentiation, for example, between bacterial/virus/parasitic diseases and occupational diseases/disorders of environmental origin/chemically induced disorders.

Of those vocabularies that can be applied to outcomes, none are suitable for the classification of all potential outcomes from clinical trials, as they provide only a partial perspective or are relevant only for specific diagnoses or fields of research. For example, physiological domains alone are categorized in some of these vocabularies, such as ICD-10 [E4], NICE [E10], UK Clinical Research Collaboration Health Research Classification System Health Categories [E11], Patient-Centered Outcomes Research Institute [E12], as well as in other ontologies relating to genetic research (e.g., the Human Phenotypic Ontology [E13], see Supplementary Table 2 on the journal's web site at www.elsevier.com). The Diagnostic and Statistical Manual of Mental Disorders (DSM, [E14]) provides a comprehensive classification system of mental disorders only. The Grid-Enabled Measures (GEM) Database [E15], an online tool for the organization of scientific measures used in behavioral, social science, and other scientific research areas, classifies measures according to physiological and methodological research areas. However, these categories do not cover the full range of potential trial outcomes, and thus fail to provide a comprehensive structure for outcome classification.

The Agency for Healthcare Research and Quality commissioned a project to determine existing methods to standardize outcome measure definitions, in order to inform the development of its Outcome Measures Framework [E16]. Their literature review identified few existing methods to categorize outcome measures, none of which are entirely relevant for our purposes. For example, the National Quality Form Quality Positioning System provides a search facility for quality measures, with search categories that extend beyond the remit of classifying trial outcomes (see Supplementary Table 2 on the journal's web site at www.elsevier.com). The Outcome Measures Framework provides a means to describe the context relating to outcome measures within patient registry entries rather than providing a comprehensive taxonomy structure for outcome classification. This system provides a conceptual framework to categorize data elements according to the characteristics of the study (in particular, of the participant, disease and treatment provider) and treatment (type and intent), as well as of the outcome (categorized as survival, disease response, events of interest, patient-reported outcomes, and health system utilization). These five main outcome classification categories do not provide a comprehensive system for classifying all trial outcomes.

The literature review identified several outcome classification systems; however, none of these provide a hierarchical structure of sufficient scope or granularity to be usefully applied to all potential trial outcomes:

(i) Wilson and Cleary [12] developed a health-related quality of life (HRQL) conceptual model rather than providing a detailed outcome taxonomy structure and excluded outcomes such as resource use or adverse events.
(ii) Patient-Reported Outcomes Measurement Information System (PROMIS, [E17]) provides a structure for classifying patient-reported measures only; outcomes collected by health care providers, and those affecting wider society, are therefore not included.
(iii) Similarly, the Nursing Outcomes Classification ([E18]) only covers outcomes relevant to nursing, thus excluding outcome domains with wider relevance, such as resource use and adverse events.
(iv) Various disease-specific classification structures provide outcome taxonomies relevant to a specific disease or condition only (National Institutes of Health Toolbox, DOMS, Neuro-QoL, ASCQ-Me, [E19]).
(v) Outcome Measures in Rheumatology (OMERACT) provides a useful structure of outcome “core areas,” with examples of domains to be included within each of these “core areas”; however, this structure is not sufficiently detailed to provide standardized classification of outcomes beyond the top “core area” level [13].
(vi) Davey et al. [14] used a data-driven approach to categorize outcomes from Cochrane Reviews into 11 categories. The disadvantage of a data-driven approach is that it potentially will not be fully comprehensive, as it may not extend beyond the collected outcomes to cover all possible trial outcomes. This structure provided a useful starting point for classification of trial outcomes but lacked a hierarchical structure; some categories are overly broad while others are too specific for classification purposes. Similarly, Smith et al. [11] grouped outcomes from Cochrane Reviews into 15 categories; however, this classification system also failed to systematically differentiate between higher level outcome types with a structured hierarchy.

### 3.2 Outcome classification systems used in COS studies

One-third (99/299) of published COS studies involved a systematic or literature review to identify relevant outcomes. Of these, 21 applied their own data-driven approach to outcome classification. Six applied an existing classification system: four studies used ICF terms, one study used a simplified version of the Wilson and Cleary model, and one study used outcome categories defined by previous authors (specifically for stroke outcomes) [15].

### 3.3 Categorization of COS outcomes

The newly proposed 38-item classification system was applied to the 299 published COS in the COMET database, where the median (range) number of outcomes per COS is 5 (1, 46). Table 2 displays the number of COS that include at least one outcome from each of the categories. Ninety-two percent (274 COS) include at least one physiological outcome, whereas only 59% (177 COS) include at least one measure of impact (HRQL or some measure of functioning). Only one-third (105, 35%) of COS explicitly call for adverse events/effects to be recorded. At least one resource use outcome was included in only 84 (28%) of COS. As expected, the breakdown according to physiological/clinical domains largely reflects the profile of diseases and conditions for which COS have been developed [5], [6], [7].

**Table 2:** Breakdown of outcomes within 299 COS in COMET database

Core area	Outcome domain	Number of COS (% of 299)
Mortality/survival	Mortality/survival	99 (33)
Physiological/clinical	Physiological/clinical (≥1)	274 (92)
	Blood and lymphatic system outcomes	9 (3)
	Cardiac outcomes	24 (8)
	Congenital, familial and genetic outcomes	1 (0.3)
	Endocrine outcomes	3 (1)
	Ear and labyrinth outcomes	3 (1)
	Eye outcomes	6 (2)
	Gastrointestinal outcomes	43 (14)
	General outcomes	57 (19)
	Hepatobiliary outcomes	6 (2)
	Immune system outcomes	6 (1)
	Infection and infestation outcomes	18 (6)
	Injury and poisoning outcomes	7 (2)
	Metabolism and nutrition outcomes	1 (0.3)
	Musculoskeletal and connective tissue outcomes	58 (19)
	Outcomes relating to neoplasms: benign, malignant and unspecified (including cysts and polyps)	33 (11)
	Nervous system outcomes	48 (17)
	Pregnancy, puerperium, and perinatal outcomes	8 (3)
	Renal and urinary outcomes	13 (4)
	Reproductive system and breast outcomes	8 (3)
	Psychiatric outcomes	23 (8)
	Respiratory, thoracic and mediastinal outcomes	32 (11)
	Skin and subcutaneous tissue outcomes	12 (4)
	Vascular outcomes	31 (10)
	
Life impact	Functioning (≥1)	128 (43)
	Physical	111 (37)
	Social	25 (8)
	Role	11 (4)
	Emotional/well-being	29 (10)
	Cognitive	21 (7)
	Global quality of life	121 (40)
	Perceived health status	0 (0)
	Delivery of care	52 (17)
	Personal circumstances	0 (0)
Resource use	Resource use (≥1)	84 (28)
	Economic	37 (12)
	Hospital	24 (8)
	Need for intervention	44 (15)
	Societal/carer burden	5 (2)
Adverse events/effects	Adverse events/effects	105 (35)

### 3.4 Categorization of systematic review outcomes

A total of 16,525 outcomes from 3,515 Cochrane Reviews have been classified according to our taxonomy to date as part of the pilot phase of the Cochrane Linked Data Project (Table 3). The majority of the annotated reviews came from the Cochrane Pregnancy and Childbirth and the Neonatal groups; a smaller set came from the Cochrane Developmental, Psychosocial and Learning Problems group. In these selected Cochrane reviews, outcomes were less commonly reported within each of the overarching outcome areas than for COS, with the exception of resource use. Less than one-quarter (831, 24%) of reviews include a measure of impact (function or quality of life, QoL) while physiological outcomes dominate, being present in 83% (2,915) of reviews annotated to date.

**Table 3:** Cochrane Linked Data Project pilot phase outcome classifications

	Number (%) of 3,515 cochrane reviews	Number (%) of 16,525 outcome classifications
Adverse events	596 (17)	951 (6)
Mortality	857 (24)	1,246 (8)
Physiological	2,915 (83)	9,820 (59)
Function/QoL	831 (24)	1,844 (11)
Delivery of care	419 (12)	493 (3)
Resource use	1,117 (32)	2,171 (13)

### 3.5 Categorization of trial outcomes

The outcomes listed in 30 studies identified from a search for randomized, phase 3 and 4 interventional studies currently recruiting participants and received by the US National Institutes of Health clinical trials registry, clinicaltrials.gov, during the first 20 days of 2017 (https://clinicaltrials.gov search terms “Randomized”, “Phase 3,4”, “Recruiting”, “Interventional Studies”, “Received from January 1, 2017 to January 20, 2017”) have been categorized in Supplementary Table 3 on the journal's web site at www.elsevier.com, demonstrating the general applicability of our ontology to trials in a trials registry.

### 3.6 Case studies

#### 3.6.1 Eczema

The taxonomy has been applied to the COS for eczema [16]. In addition, the outcomes listed in eight eczema studies identified from a search of clinicaltrials.gov (search terms “Randomized”, “Phase 3,4”, “Recruiting”, “Interventional Studies”, “Eczema”, no date restrictions) have been categorized in Supplementary Table 4 on the journal's web site at www.elsevier.com, demonstrating the general applicability of our ontology to eczema trials.

#### 3.6.2 Rheumatoid arthritis

The taxonomy has been applied to the rheumatoid arthritis (RA) COS [17]. The outcomes listed in 10 RA studies identified from a search of clinicaltrials.gov (search terms “Randomized”, “Phase 3,4”, “Recruiting”, “Interventional Studies”, “Rheumatoid Arthritis”, “Received from January 1, 2017 to January 20, 2017”) have been categorized in Supplementary Table 5 on the journal's web site at www.elsevier.com, again demonstrating the general applicability of our ontology to a particular clinical area.

## 4 Discussion

A literature review identified several health research vocabularies which extend beyond the remit of outcome classification, as well as a number of outcome classification systems. However, none of these are sufficiently comprehensive or granular for the specific purpose of classifying all potential outcomes from clinical trials with structured hierarchical differentiation between high level outcome types. We have therefore described the development of a new taxonomy that can be used for the classification of outcomes included in all trials, COS, systematic reviews, and trial registries. This classification system is based on similar top level “core areas” common to other outcome hierarchies [12], [13] but provides a more detailed taxonomy appropriate for all potential outcomes, in particular relating to physiological, functioning, and resource use domains.

Health-related quality of life (HRQL) measurement tools typically cover multiple domains (such as functioning, resource use, general physiological health, and global quality of life) and should therefore be classified within each of these domains, even when overall summary measures are reported, as we would recommend for any composite outcome. For example, see Supplementary Table 6 on the journal's web site at www.elsevier.com for the mapping between our taxonomy and the facets included in the WHOQOL-100 tool [E20].

The “global quality of life” domain in our taxonomy is reserved for specific individual questions or tools which measure the implicit composite outcome of global QoL (e.g., “How would you rate your overall quality of life?”), rather than for overall summary measures from HRQL tools covering multiple domains. Comparison of our taxonomy with the individual HRQL measures listed in Macefield [18] demonstrates that the vast majority of questions or components included in HRQL tools should be classified in domains other than global QoL. To further promote this transparency relating to the content of HRQL measures, we support the advice given by Macefield [18] that HRQL tools should be split into their individual components. For example, the Diabetes Therapy–Related Quality of Life Questionnaire can be split into various factors assessing burden on social activities and daily activities; anxiety and dissatisfaction; hypoglycemia; and treatment satisfaction [19].

The PROMIS website [E17] groups its adult and pediatric measures into profile domains in three core areas (physical, mental, and social health), along with specific domains which act as search terms to identify relevant measures. These patient-reported outcome domains can all be categorized within various physiological and functioning domains in our taxonomy, demonstrating the applicability of our taxonomy to another commonly used trial outcome resource.

Any specifically named adverse events (e.g., fatigue or pain) should be categorized under the appropriate taxonomy domain, rather than within the adverse event domain. In such cases, we would add an additional level of categorization which specifies that this outcome was reported as an adverse event. Thus, we suggest that our outcome classification system should be implemented as a two-component taxonomy, the first defining the outcome structure (as we have specified in the 38-item scale) and the second specifying whether or not the outcome is being measured as a benefit or a harm outcome. For example, the COS for colorectal cancer surgery [20] includes fecal urgency, which is a potential adverse effect of the surgery. In our system, this would be classified as a physiological outcome, under the gastrointestinal category, but a second component would identify it as an adverse outcome. In a particular example of the detailed classification of adverse events relating to total ankle arthroplasty [21], the adverse events listed can be classified within existing physiological categories, predominantly musculoskeletal and connective tissue, and infection domains.

In contrast, the adverse event domain only includes outcomes explicitly labeled as some form of unintended consequence of the intervention, such as “adverse events,” “adverse effects,” “adverse reactions,” “complications,” “toxicity,” or “sequelae”. This domain, which is not intended to include any specifically named adverse events, is important as it indicates whether or not trialists or researchers considered the need to record events that may not necessarily be prespecified ahead of time. Unless the adverse event profile is very well established for a given intervention, it is important that the incidence of all adverse events, expected or otherwise, is reported. Similarly, COS or systematic reviews that cover multiple intervention types should address the potential for unspecified adverse events.

The resource use domains in our taxonomy map well to those identified by Thorn et al. as key health economic items to be collected as part of clinical trials [22]. The 10 items in their final core set are classified under different types of care, all of which can be classified within our resource use domains: “hospital care” or “emergency care” fit within our “hospital” domain; “care at a general practice surgery, health clinic or other community setting,” and “health care at home” belong to our “societal/carer burden domain”; and “medication” fits within our “need for further intervention” domain.

We are confident that our taxonomy provides a sufficiently comprehensive basis for the categorization of outcomes included in clinical trials in general. However, we would welcome feedback from researchers applying the taxonomy in their clinical settings to demonstrate further validation of the taxonomy or to highlight any necessary changes. Note that we are not suggesting that trials or reviews should necessarily include outcomes from each of the core areas in this taxonomy. Note also that this taxonomy relates to outcomes measured at an individual-patient level (including those relating to the direct impact of the individual patient's treatment or condition on wider society, e.g., resource use or carer burden) but is not intended to cover outcomes relating to the health or functioning of wider society (e.g., family or community health). Therefore, health promotion or public health outcomes from trials of family- or community-based interventions can be classified using our taxonomy if they relate to an individual's condition or care, but not if they are measured at the family or community level.

Outcome categories within our taxonomy may be classified in even greater detail in relation to particular interventions (e.g., the classification of outcomes for childhood vaccination communication interventions [23]). Indeed, we would encourage further subdivision of each outcome domain by researchers specializing in relevant clinical or methodological areas. There may be existing taxonomies that could be used to provide finer classification within our high-level taxonomy domains; for example, the DSM could be used to classify mental disorders within the psychiatry domain.

Adoption of this classification system will facilitate literature searches; for example, if clinical trial outcomes were routinely classified according to this taxonomy, researchers would easily be able to identify clinical trials that included outcomes domains from a particular COS. A readily available taxonomy will also assist COS developers who need to categorize outcomes, for example as part of their Delphi survey, thereby speeding up the development of COS and expediting their completion and availability for use by trialists and other researchers. Application of this classification system to COS contained within the COMET database has highlighted key points to note, including that, although the COMET database relates to COS recommended for effectiveness trials, far fewer of the COS contain measures of impact (58%) than physiological outcomes (92%). Furthermore, only one-third of COS reports highlight the need to record unintended adverse consequences, and even fewer COS (29%) include any economic outcomes.

The lack of a standard taxonomy relating to trial outcomes impedes the ability to efficiently and effectively search the literature. An accepted taxonomy of outcomes would increase the reuse value of outcome data, just as MeSH terms have transformed the searchability of medical literature. The taxonomy would initially help drive to push for consistency of clinical outcome terms between clinical trials, which has been a major focus of the COMET initiative. More importantly it will allow efficient searching, reporting, and classification of clinical outcomes for the first time, thereby speeding up research activities including discovery science and “big data” approaches to extracting knowledge from published information.

In summary, the applicability of this new taxonomy has been demonstrated for the categorization of outcomes from COS, systematic reviews, and trials recorded within a clinical trial registry. Similarly, two case studies demonstrate the relevance of standardizing outcome classification to link COS, Cochrane Reviews, and trial registry entries within particular clinical areas. This taxonomy has been designed with the purpose of providing high-level differentiation between outcome domains to facilitate uniformity of outcome classification in electronic databases. We would welcome further testing of this taxonomy, and further development of subcategories to provide finer classification within each of the outcome domains is encouraged. Ongoing COS studies have used this taxonomy to classify outcomes for their initial list for a Delphi survey [E21], [E22], [E23]. We will monitor the use of the taxonomy and collate feedback, to be subsequently reported.
