# Supplementary material for: A taxonomy has been developed for outcomes in medical research to help improve knowledge discovery
Source: J Clin Epidemiol. 2018 Apr;96:84–92. doi: 10.1016/j.jclinepi.2017.12.020 (PMC5854263; doi:10.1016/j.jclinepi.2017.12.020)
Supplement: Supplementary Tables 1, 2 and 6 [file mmc1.pdf]

Supplementary Table 1 Explanation and examples of outcomes within each outcome domain

Core area	Outcome domain	Explanation
**Death**	1. Mortality/survival	Includes overall (all-cause) survival/mortality and cause-specific survival/mortality, as well as composite survival outcomes that include death (e.g. disease-free survival, progression-free survival, amputation-free survival)
**Physiological/ clinical**	**Physiological/clinical**<br><br>2. Blood and lymphatic system outcomes<br>3. Cardiac outcomes<br>4. Congenital, familial and genetic outcomes<br>5. Endocrine outcomes<br>6. Ear and labyrinth outcomes<br>7. Eye outcomes<br>8. Gastrointestinal outcomes<br>9. General outcomes<br>10. Hepatobiliary outcomes<br>11. Immune system outcomes<br>12. Infection and infestation outcomes<br>13. Injury and poisoning outcomes<br>14. Metabolism and nutrition outcomes<br>15. Musculoskeletal and connective tissue outcomes<br>16. Outcomes relating to neoplasms: benign, malignant and unspecified (including cysts and polyps)<br>17. Nervous system outcomes<br>18. Pregnancy, puerperium and perinatal outcomes<br>19. Renal and urinary outcomes<br>20. Reproductive system and breast outcomes<br>21. Psychiatric outcomes<br>22. Respiratory, thoracic and mediastinal outcomes<br>23. Skin and subcutaneous tissue outcomes	Physiological/clinical outcomes include measures of physiological function, signs and symptoms, as well as laboratory (and other scientific) measures relating to physiology, and are categorised according to the underlying cause/body system.<br><br>"General disorders" includes those affecting the whole body and cannot be attributed to a certain body system (e.g. fatigue, chills, flu like symptoms, malaise, anorexia, pain (unspecified, not associated with a particular body system), fever (not attributable to infection), anthropometric measures (e.g. weight), "global" measures, "symptoms" (not associated with a particular body system), "physical health", fitness).<br><br>Pain outcomes are categorised according to underlying cause or body system or within the "General symptoms" category (if non-specific).<br><br>Laboratory parameters (for example, from blood samples) and scientific measures (for example, pharmacokinetic outcomes) should be classified within the physiological domain that captures the reason for the assessment (rather than within the "blood and lymphatic system" category, for example).<br><br>Psychiatric outcomes include all those relating to mental health conditions and associated behaviours (e.g. addictions and behavioural problems).<br><br>Pregnancy, puerperium and perinatal domain extends to outcomes relating to

Core area	Outcome domain	Explanation
	24. Vascular outcomes	breastfeeding and weaning.<br><br>Outcomes relating to neoplasms include those related to non-solid and solid tumours.
**Life impact**	**Functioning**<br><br>25. Physical functioning<br>26. Social functioning<br>27. Role functioning<br>28. Emotional functioning/wellbeing<br>29. Cognitive functioning	**Impact outcomes**<br><br>Physical functioning: impact of disease/condition on physical activities of daily living (for example, ability to walk, independence, self-care, performance status, disability index, motor skills, sexual dysfunction. health behaviour and management)<br><br>Social functioning: impact of disease/condition on social functioning (e.g. ability to socialise, behaviour within society, communication, companionship, psychosocial development, aggression, recidivism, participation)<br><br>Role functioning: impact of disease/condition on role (e.g. ability to care for children, work status)<br><br>Emotional functioning/wellbeing: impact of disease/condition on emotions or overall wellbeing (e.g. ability to cope, worry, frustration, confidence, perceptions regarding body image and appearance, psychological status, stigma, life satisfaction, meaning and purpose, positive affect, self-esteem, self-perception and self-efficacy)<br><br>Cognitive functioning: impact of disease/condition on cognitive function (e.g. memory lapse, lack of concentration, attention); outcomes relating to knowledge, attitudes and beliefs (e.g. learning and applying knowledge, spiritual beliefs, health beliefs/knowledge)

Core area	Outcome domain	Explanation
	30. Global quality of life	Includes only implicit composite outcomes measuring global quality of life
	31. Perceived health status	Subjective ratings by the affected individual of their relative level of health
	32. Delivery of care	Includes outcomes relating to the delivery of care, including<br>• adherence/compliance<br>• patient preference<br>• tolerability/acceptability of intervention<br>• withdrawal from intervention (e.g. time to treatment failure, reason for stopping therapy)<br>• appropriateness of intervention<br>• accessibility, quality and adequacy of intervention<br>• patient/carer satisfaction (emotional rather than financial burden)<br>• process, implementation and service outcomes (e.g. overall health system performance and the impact of service provision on the users of services)
	33. Personal circumstances	Outcomes relating to patient's finances, home and environment
**Resource use**	**Resource use**<br>34. Economic<br>35. Hospital<br>36. Need for further intervention<br>37. Societal/carer burden	Economic: general outcomes (e.g. cost, resource use) not captured within other specific resource use domains<br>Hospital: outcomes relating to inpatient or day case hospital care (e.g. duration of hospital stay, admission to ICU)<br>Need for further intervention: outcomes relating to medication (e.g. concomitant medications, pain relief), surgery (e.g. caesarean delivery, time to transplantation) and other procedures (e.g. dialysis-free survival, mode of delivery)<br>Societal/carer burden: outcomes relating to financial or time implications on carer or society as a whole (e.g. need for home help, entry to institutional care, effect on family income)

Core area	Outcome domain	Explanation
**Adverse events**	38. Adverse events/effects	Includes outcomes broadly labelled as some form of unintended consequence of the intervention (e.g. adverse events/effects, adverse reactions, safety, harm, negative effects, toxicity, complications, sequelae). Specifically named adverse events should be classified within the appropriate taxonomy domain above with an additional level of categorisation which identifies that this outcome is being considered as an adverse event.

Readers are directed to the COMET website (http://www.comet-initiative.org) to access regularly updated versions of this table

Supplementary Table 2 Mapping between MedDRA, NICE, NQF QPS and HPO categorisation of physiological conditions

MedDRA	NICE	NQF: QPS[1]	HPO "Phenotypic abnormality" superclass
Blood and lymphatic system disorders<br><br>Immune system disorders	Blood and immune system conditions		Abnormality of the blood and blood-forming tissues<br><br>Abnormality of the immune system
Neoplasms benign, malignant and unspecified (including cysts and polyps)	Cancer	Cancer	Neoplasm
Cardiac disorders<br><br>Vascular disorders	Cardiovascular conditions	Cardiovascular	Abnormality of the cardiovascular system
Endocrine disorders<br><br>Metabolism and nutrition disorders	Diabetes and other endocrinal, nutritional and metabolic conditions	Endocrine	Abnormality of the endocrine system<br><br>Abnormality of metabolism/homeostasis<br><br>Growth abnormality
Gastrointestinal disorders	Digestive tract conditions<br><br>Oral and dental health	Gastrointestinal<br><br>Dental	Abnormality of the abdomen
Ear and labyrinth disorders	Ear, nose and throat conditions	Ears, Nose, Throat (ENT)	Abnormality of the ear<br><br>Abnormality of the voice<br><br>Abnormality of the head or neck (partial mapping)
Eye disorders	Eye conditions	Eye Care	Abnormality of the eye
Pregnancy, puerperium and perinatal conditions	Fertility, pregnancy and childbirth	Perinatal Health	Abnormality of prenatal development or birth
Congenital, familial and genetic disorders	Genetic conditions		
Reproductive system and breast disorders	Gynaecological conditions<br><br>Urological conditions	Gynaecology<br><br>Reproductive Health	Abnormality of the breast
Infections and infestations	Infections	Infectious Diseases	
Injury, poisoning and procedural complications	Injuries, accidents and wounds		

Renal and urinary disorders	Kidney conditions<br><br>Urological conditions	Renal	Abnormality of the genitourinary system
Hepatobiliary disorders	Liver conditions	Liver	
Psychiatric disorders	Mental health and behavioural conditions	Behavioural Health	
Musculoskeletal and connective tissue disorders	Musculoskeletal conditions	Musculoskeletal	Abnormality of connective tissue<br><br>Abnormality of the musculature<br><br>Abnormality of the skeletal system<br><br>Abnormality of limbs
Nervous system disorders	Neurological conditions	Neurology	Abnormality of the nervous system
Respiratory, thoracic and mediastinal disorders	Respiratory conditions	Respiratory	Abnormality of the thoracic cavity<br><br>Abnormality of the respiratory system
Skin and subcutaneous tissue disorders	Skin conditions		Abnormality of the integument
General disorders and administration site conditions			
[2] Investigations, Social circumstances, Surgical and medical procedures, Product issues	[2] Chronic fatigue syndrome, Multiple long-term conditions	[2] Critical Care, Palliative Care and End-of-Life Care, Surgery	
[1] Search categories for "Clinical Condition/Topic Area" only<br>[2] Extra categories (not directly mapped to other classification systems)			

Supplementary Table 6 Mapping between WHOQOL-100 and outcome taxonomy domains

WHOQOL-100 domain	Facet within WHOQOL-100 domain	Outcome taxonomy domain
	Overall QoL and General Health	Global quality of life
Physical health		
	Energy and fatigue	Physical functioning
	Pain and discomfort	Physical functioning
	Sleep and rest	Physical functioning
Psychological		
	Bodily image and appearance	Emotional functioning (emotions)
	Negative feelings	Emotional functioning
	Positive feelings	Emotional functioning
	Self-esteem	Emotional functioning
	Thinking, learning, memory and concentration	Cognitive functioning
Level of independence		
	Mobility	Physical functioning
	Activities of daily living	Physical functioning
	Dependence on medicinal substances and medical aids	Need for further intervention
	Work capacity	Role functioning
Social relationships		
	Personal relationships	Social functioning
	Social support	Societal/carer burden

	Sexual activity	Social functioning
Environment		
	Financial resources	Personal circumstances
	Freedom, physical safety and security	Emotional functioning (feelings); Personal circumstances (environment)
	Health and social care; accessibility and quality	Delivery of care
	Opportunities for acquiring new information and skills	Personal circumstances
	Participation in and opportunities for recreation/leisure activities	Social functioning (participation); Personal circumstances (opportunities)
	Physical environment (pollution/noise/traffic/climate)	Personal circumstances
	Transport	Personal circumstances
Spirituality/Religion/Personal beliefs		
	Spirituality/Religion/Personal beliefs	Cognitive functioning
