# Supplementary material for: A taxonomy has been developed for outcomes in medical research to help improve knowledge discovery
Source: J Clin Epidemiol. 2018 Apr;96:84–92. doi: 10.1016/j.jclinepi.2017.12.020 (PMC5854263; doi:10.1016/j.jclinepi.2017.12.020)
Supplement: Supplementary Table 3 [file mmc2.pdf]

Supplementary table 3: Studies registered on clinicaltrials.gov

Search terms: "Randomized" AND "Recruiting" AND Interventional Studies" AND "Phase 3,4" Studies" AND Studies received from 01/01/2017 to 01/20/2017"

Study 1: Effect of N-acetylcysteine on Alcohol and Cocaine Use Disorders: A Double-Blind Randomized Controlled Trial

Outcome classification	Outcomes
Delivery of care	Adherence: Completers (i.e. subjects who attended all study appointments) vs non-completers
Psychiatric	Abstinence by urine: Amount of positive urine tests for cocaine users
Psychiatric	Abstinence by breathalyzer: Amount of positive breathalyzer tests for alcohol users
Psychiatric	Abstinence by self report
Hospital	Days of inpatient treatment: The difference (if any) between placebo and intervention groups in the amount of inpatient treatment days
Psychiatric	Global functioning: Differences in scores of the Clinical Global Impression (CGI)
Physical functioning	Global functioning: Differences in scores of the Functioning Assessment Short Test (FAST)
Social functioning	*Global functioning: Differences in scores of the Functioning Assessment Short Test (FAST)*
Role functioning	*Global functioning: Differences in scores of the Functioning Assessment Short Test (FAST)*
Emotional functioning/wellbeing	*Global functioning: Differences in scores of the Functioning Assessment Short Test (FAST)*
Cognitive functioning	*Global functioning: Differences in scores of the Functioning Assessment Short Test (FAST)*
Psychiatric	Depressive symptoms: Differences in scores of the Beck Depression Inventory (BDI)
Psychiatric	Anxiety symptoms: Differences in scores of the Beck Anxiety Inventory (BAI)
Need for further intervention	Differences between groups regarding dosage of Brain Derived Neurotrophic Factor (BDNF)
Need for further intervention	Oxidative stress 1: Differences between groups regarding dosage of Oxidized Glutathione (GSSG)
Need for further intervention	Oxidative stress 2: Differences between groups regarding dosage of Glutathione (GSH)
Need for further intervention	Oxidative stress 3: Differences between groups regarding dosage of Glutathione Peroxidase (GPx)
Need for further intervention	Oxidative stress 4: Differences between groups regarding dosage of Catalase (CAT)
Need for further	Oxidative stress 5: Differences between groups regarding dosage of Thiobarbituric Acid Reactive Substances (TBARS)

Outcome classification	Outcomes
intervention	
Need for further intervention	Oxidative stress 6: Differences between groups regarding dosage of Superoxide Dismutase (SOD)
Need for further intervention	Oxidative stress 7: Differences between groups regarding dosage of Carbonyl
Adverse events	Adverse events: Systematic Assessment for Treatment Emergent Events (SAFTEE) application

Study 2: Pregabalin for Opiate Withdrawal Syndrome

Outcome classification	Outcomes
Delivery of care	Number of patients completed detoxification
Psychiatric	Severity of opiod withdrawal: Compares changes of severity of opioid withdrawal syndrome in two arms
Need for further intervention	Amount of Ketorolac administered
Adverse events	Adverse events
Psychiatric	Craving for opiates

Study 3: Rituximab Versus Steroids and Cyclophosphamide in the Treatment of Idiopathic Membranous Nephropathy (RI-CYCLO)

Outcome classification	Outcomes
Renal and urinary	Change in probability of complete remission
Renal and urinary	Change from baseline in proteinuria
Renal and urinary	CR (Complete Remission) or PR (Partial Remission)
Renal and urinary	Estimated Glomerular filtration rate (MDRD formula)
Renal and urinary	Serum creatinine level
Renal and urinary	Frequency of and time to relapse of nephrotic syndrome
Immune system	Frequency of auto-antibodies and its relation to therapy and proteinuria response in a subgroup of patients
Adverse events	SAEs

Study 4: A Study of Atezolizumab as Adjuvant Therapy in Participants With Renal Cell Carcinoma (RCC) at High Risk of Developing Metastasis Following Nephrectomy (IMmotion010)

**Outcome classification**	**Outcomes**
Neoplasms	Disease-Free Survival (DFS)
Survival	*Disease-Free Survival (DFS)*
Survival	Overall Survival
Neoplasms	DFS in Participants With Tumor-Infiltrating Immune Cell (IC) 1/2/3
Survival	*DFS in Participants With Tumor-Infiltrating Immune Cell (IC) 1/2/3*
Neoplasms	Disease-Specific Survival
Survival	*Disease-Specific Survival*
Neoplasms	Distant Metastasis-Free Survival
Survival	*Distant Metastasis-Free Survival*
Neoplasms	Percentage of Participants Who Are Alive and Recurrence Free at Year 3
Survival	*Percentage of Participants Who Are Alive and Recurrence Free at Year 3*
Adverse Events	Percentage of Participants With Adverse Events
Neoplasms	Maximum Serum Concentration (Cmax) of Atezolizumab
Neoplasms	Minimum Serum Concentration (Cmin) of Atezolizumab
Immune system	Percentage of Participants With Anti-Therapeutic Antibodies (ATA) to Atezolizumab

Study 5: Immunogenicity and Safety of Quadrivalent Influenza Vaccine in Children Aged 6-35 Months

**Outcome classification**	**Outcomes**
Immune system	To assess the immunogenicity after vaccination defined in the CHMP[Committee for Medical Products for Human Use] criteria for influenza vaccines: HI[hemagglutination Inhibition] derived parameters: Seroprotection rate(a lower bound of 95% CI) > 70%
Immune system	To assess the immunogenicity after vaccination defined in the CHMP[Committee for Medical Products for Human Use] criteria for influenza vaccines: HI[hemagglutination Inhibition] derived parameters: Seroconversion rate(a lower bound of 95% CI) > 40%
Immune system	To assess the immunogenicity after vaccination defined in the CHMP[Committee for Medical Products for Human Use] criteria for influenza vaccines: HI[hemagglutination Inhibition] derived parameters: GMR[geometric mean ratio, mean fold increase](a lower bound of 95% CI) > 2.5

Study 6: IMbassador250: A Study of Atezolizumab (Anti-PD-L1 Antibody) in Combination With Enzalutamide in Participants With Metastatic Castration-Resistant Prostrate Cancer (mCRPC) After Failure of an Androgen Synthesis Inhibitor And Failure of, Ineligibility For, or Refusal of a Taxane Regimen

**Outcome classification**	**Outcomes**
Survival	Overall surivival (OS)
Survival	Percentage of Participants who Survived at Month 12 and 24
Neoplasms	Time to Cancer-Related Pain Progression, as Assessed Using Modified Brief Pain Inventory (BPI)
Musculoskeletal	Time to First Symptomatic Skeletal Event (SSE)
Neoplasms	Radiographic Progression-Free Survival (rPFS)
Survival	*Radiographic Progression-Free Survival (rPFS)*
Neoplasms	Percentage of Participants Who are Radiographic Progression-Free at Month 6 and 12
Survival	*Percentage of Participants Who are Radiographic Progression-Free at Month 6 and 12*
Neoplasms	Immune-Modified rPFS, as Assessed by the Investigator
Survival	*Immune-Modified rPFS, as Assessed by the Investigator*
Neoplasms	Percentage of Participants With Greater Than (>) 50% Decrease in Prostate-Specific Antigen (PSA) From Baseline
Neoplasms	Time to PSA Progression
Neoplasms	Percentage of Participant With Objective Response, as Determined by the Investigator Through use of PCWG3 Criteria and Immune Modified Response Evaluation Criteria in Solid Tumors (mRECIST) Criteria
Adverse events	Percentage of Participants With Adverse Events
Neoplasms	Minimum Observed Serum Concentration (Cmin) of Atezolizumab
Neoplasms	Maximum Observed Serum Concentration (Cmax) of Atezolizumab
Neoplasms	Plasma Concentration of Enzalutamide
Neoplasms	Plasma Concentration of N-desmethyl Enzalutamide
Immune system	Percentage of Participants With Anti-therapeutic Antibody (ATAs) to Atezolizumab
Need for further intervention	Percentage of Participants who Required Initiation or Increase in Opiate Analgesic use for Cancer Pain
Need for further intervention	Time to Initiation or Increased Opiate Analgesic Use

Study 7: Ospemifene vs. Conjugated Estrogens in the Treatment of Postmenopausal Sexual Dysfunction

**Outcome classification**	**Outcomes**
Physical functioning	Change in Female Sexual Function Index score between groups
Reproductive	Change in pain with sex between groups
Reproductive	Change in vaginal dryness between groups
Reproductive	Change in vaginal itching between groups
Reproductive	Change in vaginal irritation between groups

Study 8: Efficacy and Safety of Hou Gu Mi Xi in Patients With Spleen Qi Deficiency and Mild Gastrointestinal Disorder

Outcome classification	Outcomes
Gastrointestinal	Change from baseline to 52 weeks in scores of Spleen Qi Deficiency Symptoms Grading and Quantifying Scale (Units on a scale)
Gastrointestinal	Change from baseline to 2, 4, 8, 26, 78 and 104 weeks in scores of Spleen Qi Deficiency Symptoms Grading and Quantifying Scale (Units on a scale)
Gastrointestinal	Change from baseline to 8, 26, 78 and 104 weeks in Gastrin-17 (ng/L)
Gastrointestinal	Quantitative helicobacter pylori
General symptoms	Changes from baseline to 2, 4, 8, 26, 52, 78 and 104 weeks in body weight (kg)
General symptoms	Changes from baseline to 2, 4, 8, 26, 52, 78 and 104 weeks in body mass index (kg/m2)
Cardiac	Changes from baseline to 2, 4, 8, 26, 52, 78 and 104 weeks in systolic blood pressure (mmHg)
Cardiac	Changes from baseline to 2, 4, 8, 26, 52, 78 and 104 weeks in diastolic blood pressure (mmHg)
Gastrointestinal	Quantitative results of gastroscopy
Adverse events	Number of patients with adverse events
Adverse events	Number of patients with severe adverse events
Adverse events	Number of patients with drug-related adverse events
Delivery of care	Number of patients withdrawn due to adverse events
Cardiac	Incidence of abnormal electrocardiogram
Hepatobiliary	Changes from baseline to 8, 26, 52, 78 and 104 weeks in alanine transaminase (U/L)
Hepatobiliary	Changes from baseline to 8, 26, 52, 78 and 104 weeks in aspartate aminotransferase (U/L)
Hepatobiliary	Changes from baseline to 8, 26, 52, 78 and 104 weeks in total bilirubin (µmol/L)
Hepatobiliary	Changes from baseline to 8, 26, 52, 78 and 104 weeks in direct bilirubin (µmol/L)
Hepatobiliary	Changes from baseline to 8, 26, 52, 78 and 104 weeks in indirect bilirubin (µmol/L)
Renal and urinary	Changes from baseline to 8, 26, 52, 78 and 104 weeks in serum creatinine (µmol/L)

Study 9: Efficacy and Safety of Hou Gu Mi Xi in Patients With Spleen Qi Deficiency and Radical Gastrectomy for Gastric Cancer

Outcome classification	Outcomes
Gastrointestinal	Changes from baseline to 52 weeks in scores of Spleen Qi Deficiency Symptoms Grading and Quantifying Scale
Gastrointestinal	Changes from baseline to 2, 4, 8, 26, 78 and 104 weeks in scores of Spleen Qi Deficiency Symptoms
Physical functioning	Changes from baseline to 2, 4, 8, 26, 78 and 104 weeks in scores of Short Form 36 (SF-36) Physical Component Summary (PCS)
Emotional functioning/wellbeing	Changes from baseline to 2, 4, 8, 26, 78 and 104 weeks in scores of Short Form 36 (SF-36) Mental Component Summary (MCS)
Neoplasms	Changes from baseline to 2, 4, 8, 26, 78 and 104 weeks in scores of Eastern Cooperative Oncology Group (ECOG) Performance Status
Cardiac	Changes from baseline to 2, 4, 8, 26, 52, 78 and 104 weeks in systolic blood pressure (mmHg)
Cardiac	Changes from baseline to 2, 4, 8, 26, 52, 78 and 104 weeks in diastolic blood pressure (mmHg)
General symptoms	Changes from baseline to 2, 4, 8, 26, 52, 78 and 104 weeks in body weight (kg)
General symptoms	Changes from baseline to 2, 4, 8, 26, 52, 78 and 104 weeks in body mass index (kg/m2)
Adverse events	Number of patients with adverse events
Adverse events	Number of patients with severe adverse events
Adverse events	Number of patients with drug-related adverse events
Delivery of care	Number of patients withdrawn due to adverse events
Cardiac	Incidence of abnormal electrocardiogram
Hepatobiliary	Changes from baseline to 8, 26, 52, 78 and 104 weeks in alanine transaminase (U/L)
Hepatobiliary	Changes from baseline to 8, 26, 52, 78 and 104 weeks in aspartate aminotransferase (U/L)
Hepatobiliary	Changes from baseline to 8, 26, 52, 78 and 104 weeks in total bilirubin (µmol/L)
Hepatobiliary	Changes from baseline to 8, 26, 52, 78 and 104 weeks in direct bilirubin (µmol/L)
Hepatobiliary	Changes from baseline to 8, 26, 52, 78 and 104 weeks in indirect bilirubin (µmol/L)
Renal and urinary	Changes from baseline to 8, 26, 52, 78 and 104 weeks in serum creatinine (µmol/L)
Renal and urinary	Changes from baseline to 8, 26, 52, 78 and 104 weeks in urea nitrogen (mmol/L)
Blood and lymphatic	Changes from baseline to 8, 26, 78 and 104 weeks in prothrombin time (second)
Blood and lymphatic	Changes from baseline to 8, 26, 78 and 104 weeks in activated partial thromboplastin time
Blood and lymphatic	Changes from baseline to 8, 26, 78 and 104 weeks in thrombin time (second)
Blood and lymphatic	Changes from baseline to 8, 26, 78 and 104 weeks in fibrinogen (g/L)

Study 10: Sequential Neo-adjuvant Chemotherapy Followed by Capecitabine Vs. Conventional Adjuvant Chemotherapy in Breast Cancer (NACVCAC)

**Outcome classification**	**Outcomes**
Neoplasms	Disease-free survival (DFS)
Survival	*Disease-free survival (DFS)*
Survival	Overall survival(OS)

Study 11: Enamel Matrix Proteins in the Treatment of Intrabony Defects in Patients With Aggressive and Chronic Periodontitis

**Outcome classification**	**Outcomes**
Gastrointestinal	Relative Clinical Attachment Level

Study 12: Prophylactic Oral Antibiotics on Sinonasal Outcomes Following Endoscopic Transsphenoidal Surgery for Pituitary Lesions (POET)

**Outcome classification**	**Outcomes**
Physical functioning	Change in quality of life from baseline - Anterior Skull Base Nasal Inventory 12 (ASK Nasal-12)
Physical functioning	Change in quality of life from baseline - Sino-Nasal Outcome Test (SNOT-22)
Emotional functioning/wellbeing	*Change in quality of life from baseline - Sino-Nasal Outcome Test (SNOT-22)*
Cognitive functioning	*Change in quality of life from baseline - Sino-Nasal Outcome Test (SNOT-22)*
Infections	Incidence of Acute Bacterial Sinusitis
Ear and labyrinth	Changes in endoscopic appearances using Postoperative Debridement Scoring Sheet (modified Lund-Kennedy score)
Infections	Sinusitis and antibiotic resistance as evidenced by nasal congestion
Delivery of care	*Sinusitis and antibiotic resistance as evidenced by nasal congestion*
Infections	Sinusitis and antibiotic resistance as evidenced by purulent discharge
Delivery of care	*Sinusitis and antibiotic resistance as evidenced by purulent discharge*
Infections	Sinusitis and antibiotic resistance as evidenced by pressure
Delivery of care	*Sinusitis and antibiotic resistance as evidenced by pressure*
Infections	Sinusitis and antibiotic resistance as evidenced by pain
Delivery of care	*Sinusitis and antibiotic resistance as evidenced by pain*
Infections	Sinusitis and antibiotic resistance as evidenced headache
Delivery of care	*Sinusitis and antibiotic resistance as evidenced headache*

Study 13: Fertility Preservation Using Tamoxifen and Letrozole in Estrogen Sensitive Tumors Trial (TALES)

Outcome classification	Outcomes
Reproductive	Mature Oocyte Yield

Study 14: Effect of Immunonutrition on Septic Complications After Roux-en-Y Gastric Bypass

Outcome classification	Outcomes
Gastrointestinal	Septic complications
Blood and lymphatic	Acute phase reactants

Study 15: The Role of Platelet Rich Plasma Towards the Repair of Pelvic Floor Muscle Damage in Primipara

Outcome classification	Outcomes
Musculoskeletal	Change from baseline (third trimester) lower hiatal area during contraction measured using pelvic floor USG at 7 days post partum
Musculoskeletal	Change from baseline (third trimester) lower hiatal area during contraction measured using pelvic floor USG at 40 days post partum
Musculoskeletal	Change from baseline (third trimester) lower hiatal area during contraction measured using pelvic floor USG at 3 months post partum
Musculoskeletal	Change from baseline (third trimester) lower hiatal area measured using pelvic floor USG during valsava at 7 days post partum
Musculoskeletal	Change from baseline (third trimester) lower hiatal area during valsava measured using pelvic floor USG at 40 days post partum
Musculoskeletal	Change from baseline (third trimester) lower hiatal area during valsava measured using pelvic floor USG at 3 months post partum
Musculoskeletal	Pelvic Floor Muscle Contraction [Time Frame: Third trimester and 7 days post partum]
Musculoskeletal	Pelvic Floor Muscle Contraction [Time Frame: Third trimester and 40 days post partum]
Musculoskeletal	Pelvic Floor Muscle Contraction [Time Frame: Third trimester and 3 months post partum]
Blood and lymphatic	Creatine Kinase
Blood and lymphatic	IGF-1
Blood and lymphatic	myoD
General symptoms	Pain [Time Frame: before labor, 7 days post partum, 40 days post partum, and 3 months post partum]

Study 16: Single-shot Pectoral Plane(PECs) Block Versus Continuous Local Anaesthetic Infusion Analgesia or Both PECS Block and Local Anaesthetic Infusion After Breast Surgery: A Randomised, Double-blind, Non-inferiority Trial

Outcome classification	Outcomes
General symptoms	Area under curve of NRS (numerical rating score) pain score versus time
Need for further intervention	Oxycodone consumption over a 24hour period postoperatively
Gastrointestinal	Postoperative nausea and vomiting
General symptoms	Postoperative sedation score
Reproductive	Chronic persistent breast pain at 3 months by telephone interview using Short Form McGill questionnaire

Study 17: Cytoreductive Surgery Combined With HIPEC and Chemotherapy for Gastric Cancer With Peritoneal Metastasis

Outcome classification	Outcomes
Survival	Median survival time
Survival	2-year overall survival rate
Neoplasms	Progression free survival rate
Survival	*Progression free survival rate*
Adverse events	Morbidity and mortality [early (up to 30 days after intervention) and late (beyond 30 days after intervention) complications ranked from grade 0-5 according to CTCAE V4.0]
Survival	*Morbidity and mortality [early (up to 30 days after intervention) and late (beyond 30 days after intervention) complications ranked from grade 0-5 according to CTCAE V4.0]*
Physical functioning	Quality of life(site-specific module for gastric cancer) [Evaluated according to STO 22]
Emotional functioning/wellbeing	*Quality of life(site-specific module for gastric cancer) [Evaluated according to STO 22]*
Global quality of life	Quality of life [Evaluated according to EORTC QLQ-30]
Physical functioning	*Quality of life [Evaluated according to EORTC QLQ-30]*
Emotional functioning/wellbeing	*Quality of life [Evaluated according to EORTC QLQ-30]*
Role functioning	*Quality of life [Evaluated according to EORTC QLQ-30]*
Cognitive functioning	*Quality of life [Evaluated according to EORTC QLQ-30]*
Social functioning	*Quality of life [Evaluated according to EORTC QLQ-30]*
Personal circumstances	*Quality of life [Evaluated according to EORTC QLQ-30]*
Neoplasms	CTC (circulating tumor cell) alteration
Neoplasms	ctDNA (circulating tumor DNA) alteration
Neoplasms	Molecular biomarker alteration [Molecular biomarker includes TP53, BAI1, THSD1, ARID2, KIAA2022 , ZNF721, NT5E, PDE10A,CA1, NUMB, NBN, ZFYVE16 and NCAM1 according to the whole-exome sequencing results.]

Study 18: Oxycodone vs. Fentanyl in Early Postoperative Pain After Total Hip Replacement

Outcome classification	Outcomes
General symptoms	Postoperative pain measurement using Numeric Rating Scale(NRS)
Need for further intervention	Additional doses of fentanyl for pain management
Adverse events	Adverse effect

Study 19: Effect of Transportation Method on Preoperative Anxiety in Children

Outcome classification	Outcomes
Emotional functioning/wellbeing	Preoperative anxiety score using the Modified Yale Preoperative Anxiety Scale

Study 20: Dose-response, Safety and Efficacy of Oral Semaglutide Versus Placebo and Versus Liraglutide, All as Monotherapy in Japanese Subjects With Type 2 Diabetes (PIONEER 9)

Outcome classification	Outcomes
Endocrine	Change in HbA1c [Time Frame: week 0, week 26]
Endocrine	Change in HbA1c [Time Frame: week 0, week 52]
Endocrine	Change in Fasting plasma glucose
General symptoms	Change in Body weight (kg)
Endocrine	Number of subjects achieving Yes/No in level of glycosylated haemoglobin (HbA1c) below 7% (53 mmol/mol)

Study 21: The Optimal Neck Treatments Strategy of Early Oral Cancer Based on Adverse Pathological Factor

Outcome classification	Outcomes
Neoplasms	Neck control rates
Neoplasms	Disease-free survival [Various Time Frames: 1 year, 2 years, 3 years, 5 years]
Survival	*Disease-free survival [Various Time Frames: 1 year, 2 years, 3 years, 5 years]*
Survival	Overall survival [Time Frame: 3 years, 5 years]

Study 22: A Study of the Efficacy and Safety of TACI-antibody Fusion Protein Injection (RC18) in Subjects With Inadequate Response to MTX Due to Treat Moderate and Severe Rheumatoid Arthritis

Outcome classification	Outcomes
Musculoskeletal	The proportion of patients in each group reached ACR20 24 weeks for visits
Musculoskeletal	Percentage of Participants Achieving American College of Rheumatology ACR50 and ACR70 Responses at week 24
Musculoskeletal	Percentage of Participants Achieving Low Disease Activity and clinical remission. (DAS28 ≤3.20 and DAS28 < 2.6)
Musculoskeletal	Percentage of Participants Achieving American College of Rheumatology ACR50 and ACR70 Responses at week 12 or week 24
Musculoskeletal	Sharp Score Relative Change from Baseline at Week 24
Musculoskeletal	Percentage of Participants With American College of Rheumatology 20% ,50% and 70% (ACR20, ACR50 and ACR70) Response
Musculoskeletal	Change From Baseline in Joint Space Narrowing and Erosions at week 24 and week 48

Study 23: Effect of Dexmedetomidine for Postoperative Intravenous Patient Controlled Analgesia

Outcome classification	Outcomes
Need for further intervention	Dezocine consumption by patient-controlled analgesia
General symptoms	Change in pain score
General symptoms	Change in ramsay sedation score
Gastrointestinal	The incidence rates of postoperative nausea and vomiting

Study 24: Study of the QOL Evaluation of Trelagliptin in Patients With Type 2 Diabetes Mellitus

**Outcome classification**	**Outcomes**
Physical functioning	Change from baseline (Week 0) in total score for all question items in the Diabetes Therapy Related-QOL (DTR-QOL) Questionnaire at Week12
Social functioning	*Change from baseline (Week 0) in total score for all question items in the Diabetes Therapy Related -QOL (DTR-QOL) Questionnaire at Week 12*
Emotional functioning/wellbeing	*Change from baseline (Week 0) in total score for all question items in the Diabetes Therapy Related -QOL (DTR-QOL) Questionnaire at Week 12*
Delivery of care	*Change from baseline (Week 0) in total score for all question items in the Diabetes Therapy Related -QOL (DTR-QOL) Questionnaire at Week 12*
Endocrine	*Change from baseline (Week 0) in total score for all question items in the Diabetes Therapy Related -QOL (DTR-QOL) Questionnaire at Week 12*
Social functioning	DTR-QOL Questionnaire [Factor 1: Burden on social activities and daily activities] at each assessment time point
Physical functioning	*DTR-QOL Questionnaire [Factor 1: Burden on social activities and daily activities] at each assessment time point*
Emotional functioning/wellbeing	DTR-QOL Questionnaire [Factor 2: Anxiety and dissatisfaction with treatment] at each assessment time point
Delivery of care	*DTR-QOL Questionnaire [Factor 2: Anxiety and dissatisfaction with treatment] at each assessment time point*
Emotional functioning/wellbeing	DTR-QOL Questionnaire [Factor 3: Hypoglycemia] at each assessment time point
Endocrine	*DTR-QOL Questionnaire [Factor 3: Hypoglycemia] at each assessment time point*
Delivery of care	DTR-QOL Questionnaire [Factor 4: Treatment satisfaction] at each assessment time point
Physical functioning	Total score for all questions in the DTR-QOL Questionnaire at each assessment time point
Social functioning	*Total score for all questions in the DTR-QOL Questionnaire at each assessment time point*
Physical functioning	*Total score for all questions in the DTR-QOL Questionnaire at each assessment time point*
Emotional functioning/wellbeing	*Total score for all questions in the DTR-QOL Questionnaire at each assessment time point*
Delivery of care	*Total score for all questions in the DTR-QOL Questionnaire at each assessment time point*
Endocrine	*Total score for all questions in the DTR-QOL Questionnaire at each assessment time point*
Delivery of care	Diabetes Treatment Satisfaction Questionnaire (DTSQ) [6 questions about treatment satisfaction and 2 questions regarding blood sugar level.]
Endocrine	Diabetes Treatment Satisfaction Questionnaire (DTSQ) [6 questions about treatment satisfaction and 2 questions regarding blood sugar level.]
Adverse events	Number of participants who had one or more adverse events
Endocrine	Number of participants who had one or more hypoglycemia
Hospital	Duration of hospitalization for type 2 diabetes (excluding educational hospitalization without worsening of diabetes)
Hospital	Number of participants with hospitalization for type 2 diabetes (excluding educational hospitalization without worsening of diabetes)

Study 25: Melatonin Use in the Intensive Care Elderly Population (MICE)

**Outcome classification**	**Outcomes**
General symptoms	Delirium
Need for further intervention	Use of Antipsychotic Medications
General symptoms	Average RASS Score *(Richmond Agitation-Sedation Scale)*
Hospital	ICU days
Hospital	Hospital Days
Survival	30 day mortality
Survival	90 day mortality

Study 26: General Plus Spinal Anesthesia and General Anesthesia Alone on Right Ventricular Function

**Outcome classification**	**Outcomes**
Cardiovascular	Improvement of right ventricular function defined by 20% increase in the TAPSE in the study group when compared to the control group
Cardiovascular	Pulmonary vascular resistance
Cardiovascular	Left ventricular ejection fraction
Cardiovascular	Right ventricular myocardial performance index
Need for further intervention	Mechanical ventilation duration
Hospital	Intensive care unit stay

Study 27: Liraglutide as an Additional Treatment to Insulin in Patients With Autoimmune Diabetes Mellitus

Outcome classification	Outcomes
Endocrine	Mean amplitude of glycemic excursions (MAGE)
Endocrine	Change in HbA1C
Endocrine	Change in C-peptide
Need for further intervention	Change in insulin dose
Endocrine	Hyperglycemic and hypoglycemic events
Physical functioning	Life quality evaluation (Number of subjects with disturbance of emotion, sleep, resting or energy.)
Emotional functioning	*Life quality evaluation (Number of subjects with disturbance of emotion, sleep, resting or energy.)*

Study 28: Efficacy Study of Oral Nicorandil on Improving Microvascular Function in Female Non-obstructive Coronary Artery Disease (CAD) Subjects (SPET)

**Outcome classification**	**Outcomes**
Cardiovascular	Change from Baseline in Myocardial Blood Flow Reserve (MFR) by stress PET at Week 12
Cardiovascular	Change from Baseline in Myocardial Blood Flow (MBF) by rest PET at Week 12
Cardiovascular	Change from Baseline in MBF by stress PET at Week 12
Cardiovascular	Change from Baseline in Ejection Fraction at Week 12
Cardiovascular	Change from Baseline in Left Ventricular End-Systolic Dimension (LVESD) at Week 12
Cardiovascular	Change from Baseline in left ventricular wall thickness at Week 12
Cardiovascular	Change from Baseline in cardiac diastolic function: early [E] to late [A] ventricular filling velocities (E/A) ratio at Week 12
Physical functioning	Change from Baseline in Seattle Angina Questionnaire(SAQ) score at Week 12
Cognitive functioning	*Change from Baseline in Seattle Angina Questionnaire(SAQ) score at Week 12*
Cardiovascular	*Change from Baseline in Seattle Angina Questionnaire(SAQ) score at Week 12*
Delivery of care	*Change from Baseline in Seattle Angina Questionnaire(SAQ) score at Week 12*

Study 29: Letrozole Versus Laparoscopic Ovarian Drilling in Polycystic Ovary Syndrome

**Outcome classification**	**Outcomes**
Gynaecological	The ovulation rate
Gynaecological	The mid-cyclic endometrial thickness

Study 30: Does Simethicone Improve Operative Field in Gynecological Operations

**Outcome classification**	**Outcomes**
Digestive tract	The degree of small & large bowel preparation
