# Supplementary material for: A taxonomy has been developed for outcomes in medical research to help improve knowledge discovery
Source: J Clin Epidemiol. 2018 Apr;96:84–92. doi: 10.1016/j.jclinepi.2017.12.020 (PMC5854263; doi:10.1016/j.jclinepi.2017.12.020)
Supplement: Supplementary Table 4 [file mmc3.pdf]

Supplementary table 4: Eczema studies registered on clinicaltrials.gov

Search terms: “Randomized" AND "Recruiting" AND “Interventional Studies" "Eczema" "Phase 3,4"

Study 1: Alitretinoin vs Azathioprine in Severe Non-hyperkeratotic Hand Eczema (ALIAZ)

Outcome classification	Outcomes
Skin	Response to treatment/hand eczema severity (Photoguide) [Time Frame: 24 weeks (end of treatment)]
Skin	Response to treatment/hand eczema severity (Photoguide) [Time Frame: 12 weeks]
Skin	Response to treatment/hand eczema severity (Hand Eczema Severity Index, HECSI) [Time Frame: Week 4, 8, 12, 24]
Skin	Time to response [Time Frame: Week 4, 8, 12, 24]
Skin	Patient reported improvement (Patient Global Assessment, PaGA) [Time Frame: Week 12 and 24]
Adverse events	Safety and tolerability (adverse events) [Time Frame: Up to 24 weeks]
Economic	Cost-utility. QALY's: registered direct/indirect costs, combined with EQ-5D outcome [Time Frame: Week 12 and 24]
Economic	Cost-effectiveness: registered direct/indirect costs combined with the primary and secondary effectiveness outcomes (Photoguide/HECSI) [Time Frame: Week 12 and 24]
Global quality of life	Quality of Life: questionnaire. [Time Frame: Week 12 and 24]

Study 2: Study of Generic Pimecrolimus Cream, 1% in the Treatment of Mild to Moderate Atopic Dermatitis

Outcome classification	Outcomes
Skin	The proportion of subjects in each treatment group with treatment success (i.e., a grade of clear or almost clear; a score of 0 or 1, within the treatment area) based on the IGA of Disease Severity at the end of treatment (Visit 3; Day 15 ± 3). [Time Frame: Day 15]
Skin	Change in severity score from baseline to Visit 3 of the four individual signs and symptoms of AD (i.e., erythema, induration/papulation, lichenification and pruritus). [Time Frame: Day 15]

Study 3: Efficacy and Safety of Runzao Zhiyang Capsule to Treat Chronic Eczema

Outcome classification	Outcomes
Skin	The efficiency (The number of cured cases and cases with marked effect) of disease: Using EASI score decline rate to judge. The decline rate of EASI = (before treatment EASI- After treatment EASI)/ before treatment EASI×100% [Time Frame: 0,4 weeks]
Skin	The decrease rate of EASI in each visit compare with baseline [Time Frame: 0,2,4,8,12 weeks]
Skin	Changes in the degree of pruritus （test by visual analogue scale） in the follow-up visits compared with baseline [Time Frame: 0,2,4,8,12 weeks]
Physical functioning	DLQI (Dermatology Life Quality Index) score changes during the follow-up visits compared with baseline. [Time Frame: 0,2,4,8,12 weeks]
Role functioning	DLQI (Dermatology Life Quality Index) score changes during the follow-up visits compared with baseline. [Time Frame: 0,2,4,8,12 weeks]
Social functioning	DLQI (Dermatology Life Quality Index) score changes during the follow-up visits compared with baseline. [Time Frame: 0,2,4,8,12 weeks]
Emotional functioning/wellbeing	DLQI (Dermatology Life Quality Index) score changes during the follow-up visits compared with baseline. [Time Frame: 0,2,4,8,12 weeks]
Skin	The proportion of patients (EASI score greater than 10% of the patients before treatment) [Time Frame: 8,12 weeks]
Skin	EASI score [Time Frame: 8,12 weeks]
Adverse events	Safety assessments will be based on adverse event reports. [Time Frame: 2,4,8,12 weeks]
Adverse events	Safety assessments will be based on electrocardiogram, physical examinations, and clinical laboratory tests. [Time Frame: 0,2,4,8,12 weeks]

Study 4: Clinical Study to Evaluate the Efficacy and Safety of DSXS With Atopic Dermatitis

Outcome classification	Outcomes
Skin	The proportion of patients in each treatment group that have clinical success [Time Frame: 28 days]

Study 5: CAPTAIN-AD: Clinical Study of AmorePacific's TRPV1 Antagonist in Atopic Dermatitis

Outcome classification	Outcomes
Skin	Success rate of Investigator's Global Assessment (IGA) [Time Frame: 8 weeks]
Skin	Change of Investigator's Global Assessment (IGA) score [Time Frame: 8 weeks]
Skin	Success rate of ≥2-grade Investigator's Global Assessment (IGA) [Time Frame: 8 weeks]
Skin	Percent of change in Eczema Area and Severity Index (EASI) [Time Frame: 8 weeks]

Study 6: Development of a Personalized Immune-modulating Therapy for Patients With Atopic Dermatitis

Outcome classification	Outcomes
Skin	Change in EASI index [Time Frame: baseline to week 16]
Skin	Change in EASI index [Time Frame: baseline to week 8]
Skin	Change in SCORAD values [Time Frame: baseline to week 16]
Skin	Change in IGA [Time Frame: baseline to week 16]
Physical functioning	Change in DLQI index [Time Frame: baseline to week 16]
Role functioning	*Change in DLQI index [Time Frame: baseline to week 16]*
Social functioning	*Change in DLQI index [Time Frame: baseline to week 16]*
Emotional functioning/wellbeing	*Change in DLQI index [Time Frame: baseline to week 16]*

Study 7: Role of Anti-IgE in Severe Childhood Eczema (ADAPT)

Outcome classification	Outcomes
Skin	Improvement in atopic eczema [Time Frame: 24 weeks after treatment commences]

Study 8: Can Vitamin D Supplementation in Infants Prevent Food Allergy in the First Year of Life? The VITALITY Trial (VITALITY)

Outcome classification	Outcomes
Skin	The prevalence and severity of challenge-proven food allergy in study participants with positive skin prick tests (SPT) [Time Frame: at age 12 months]
Skin	Prevalence of food sensitisation (positive skin prick) test [Time Frame: age 12 months]
Skin	Doctor diagnosed eczema during the first postnatal year [Time Frame: age 12 months]
Skin	Vitamin D insufficiency [Time Frame: at age 12 months]
