# Supplementary material for: A taxonomy has been developed for outcomes in medical research to help improve knowledge discovery
Source: J Clin Epidemiol. 2018 Apr;96:84–92. doi: 10.1016/j.jclinepi.2017.12.020 (PMC5854263; doi:10.1016/j.jclinepi.2017.12.020)
Supplement: Supplementary Table 5 [file mmc4.pdf]

Supplementary table 5: Rheumatoid Arthritis studies registered on clinicaltrials.gov

Search terms: “Randomized" AND "Recruiting" AND Interventional Studies" "Rheumatoid Arthritis" "Phase 3,4" "Studies received from 07/01/2016 to 01/20/2017”

Study 1: Methotrexate Withdrawal Study of Tofacitinib Modified Release Formulation in Subjects With Rheumatoid Arthritis

Outcome classification	Outcomes
Musculoskeletal	Change in DAS28-4 (ESR) score from randomization (at week 24) to the end of double-blind MTX withdrawal phase (at week 48) [Time Frame: week 48]
Musculoskeletal	Change in the DAS28-4(ESR) from week 24 to week 36 [Time Frame: week 36]
Musculoskeletal	Change in the DAS28-4 (CRP) from week 24 to week 48 [Time Frame: week 48]
Musculoskeletal	Change in the CDAI from week 24 to week 48 [Time Frame: week 48]
Musculoskeletal	Change in SDAI from week 24 to week 48 [Time Frame: week 48]
Musculoskeletal	Change in the DAS28-4 (CRP) from week 24 to week 36 [Time Frame: week 36]
Musculoskeletal	Change in the CDAI from week 24 to week 36 [Time Frame: week 36]
Musculoskeletal	Change in the SDAI from week 24 to week 36 [Time Frame: week 36]
Musculoskeletal	LDA as assessed by DAS28-4(ESR) <3.2 at week 48 [Time Frame: week 48]
Musculoskeletal	LDA as assessed by DAS28-4(CRP) <3.2 at week 48 [Time Frame: week 48]
Musculoskeletal	LDA as assessed by CDAI<=10 at week 48 [Time Frame: week 48]
Musculoskeletal	LDA as assessed by SDAI<=11 at week 48 [Time Frame: week 48]
Musculoskeletal	LDA as assessed by DAS28-4(ESR) <3.2 at week 36 [Time Frame: week 36]
Musculoskeletal	LDA as assessed by DAS28-4(CRP) <3.2 at week 36 [Time Frame: week 36]
Musculoskeletal	LDA as assessed by CDAI<=10 at week 36 [Time Frame: week 36]
Musculoskeletal	LDA as assessed by SDAI<=11 at week 36 [Time Frame: week 36]
Musculoskeletal	Remission as assessed by ACR-EULAR Boolean remission criteria at week 48 [Time Frame: week 48]
Musculoskeletal	Remission as assessed by DAS28-4 (ESR)<2.6 at week 48 [Time Frame: week 48]
Musculoskeletal	Remission as assessed by DAS28-4 (CRP)<2.6 at week 48 [Time Frame: week 48]
Musculoskeletal	Remission as assessed by CDAI≤2.8 at week 48 [Time Frame: week 48]
Musculoskeletal	Remission as assessed by SDAI≤3.3 at week 48 [Time Frame: week 48]
Musculoskeletal	Remission as assessed by ACR-EULAR Boolean remission criteria at week 36 [Time Frame: week 36]
Musculoskeletal	Remission as assessed by DAS28-4 (ESR)<2.6 at week 36 [Time Frame: week 36]
Musculoskeletal	Remission as assessed by DAS28-4 (CRP)<2.6 at week 36 [Time Frame: week 36]
Musculoskeletal	Remission as assessed by CDAI≤2.8 at week 36 [Time Frame: week 36]
Musculoskeletal	Remission as assessed by SDAI≤3.3 at week 36 [Time Frame: week 36]

Outcome classification	Outcomes
Musculoskeletal	ACR20 response at week 48 [Time Frame: week 48]
Musculoskeletal	ACR50 response at week 48 [Time Frame: week 48]
Musculoskeletal	ACR70 response at week 48 [Time Frame: week 48]
Musculoskeletal	ACR20 response at week 36 [Time Frame: week 36]
Musculoskeletal	ACR50 response at week 36 [Time Frame: week 36]
Musculoskeletal	ACR70 response at week 36 [Time Frame: week 36]
Physical functioning	Change in the HAQ-DI from week 24 to week 48 [Time Frame: week 48]
Physical functioning	Change in the SF-36 (8 domain scores and 2 component scores) from week 24 to week 48 [Time Frame: week 48]
Social functioning	*Change in the SF-36 (8 domain scores and 2 component scores) from week 24 to week 48 [Time Frame: week 48]*
Role functioning	*Change in the SF-36 (8 domain scores and 2 component scores) from week 24 to week 48 [Time Frame: week 48]*
Emotional functioning/ wellbeing	*Change in the SF-36 (8 domain scores and 2 component scores) from week 24 to week 48 [Time Frame: week 48]*
Physical functioning	Change in the WPAI score from week 24 to week 48 [Time Frame: week 48]
Role functioning	*Change in the WPAI score from week 24 to week 48 [Time Frame: week 48]*
Physical functioning	Change in the EuroQol EQ-5D score from week 24 to week 48 [Time Frame: week 48]
Emotional functioning/wellbeing	*Change in the EuroQol EQ-5D score from week 24 to week 48 [Time Frame: week 48]*
Physical functioning	Change in the FACIT-Fatigue scale score from week 24 to week 48 [Time Frame: week 48]
Social functioning	*Change in the FACIT-Fatigue scale score from week 24 to week 48 [Time Frame: week 48]*
Physical functioning	Change in the HAQ-DI from week 24 to week 36 [Time Frame: week 36]
Physical functioning	Change in the SF-36 (8 domain scores and 2 component scores) from week 24 to week 36 [Time Frame: week 36]
Social functioning	*Change in the SF-36 (8 domain scores and 2 component scores) from week 24 to week 48 [Time Frame: week 48]*
Role functioning	*Change in the SF-36 (8 domain scores and 2 component scores) from week 24 to week 48 [Time Frame: week 48]*
Emotional functioning/ wellbeing	*Change in the SF-36 (8 domain scores and 2 component scores) from week 24 to week 48 [Time Frame: week 48]*
Physical functioning	Change in the WPAI score from week 24 to week 36 [Time Frame: week 36]
Role functioning	*Change in the WPAI score from week 24 to week 48 [Time Frame: week 48]*
Physical functioning	Change in the EuroQol EQ-5D score from week 24 to week 36 [Time Frame: week 36]
Emotional functioning/ wellbeing	*Change in the EuroQol EQ-5D score from week 24 to week 48 [Time Frame: week 48]*
Physical functioning	Change in the FACIT-Fatigue scale score from week 24 to week 36 [Time Frame: week 36]
Social functioning	*Change in the FACIT-Fatigue scale score from week 24 to week 48 [Time Frame: week 48]*
Physical functioning	HAQ-DI response (ie, decrease of at least 0.22) at week 48 [Time Frame: week 48]
Physical functioning	HAQ-DI response (ie, decrease of at least 0.22) at week 36 [Time Frame: week 36]

Study 2: Filgotinib Alone and in Combination With Methotrexate (MTX) in Adults With Moderately to Severely Active Rheumatoid Arthritis Who Are Naive to MTX Therapy

**Outcome classification**	**Outcomes**
Musculoskeletal	Proportion of Participants who Achieve an American College of Rheumatology (ACR) 20% Improvement (ACR20) Response at Week 24 [Time Frame: Week 24]
Physical functioning	Change from Baseline in the Health Assessment Questionnaire - Disability Index (HAQ-DI) Score at Week 24 [Time Frame: Week 24]
Musculoskeletal	Proportion of Participants who Achieve Disease Activity Score based on 28 joints (DAS28) (C-reactive protein (CRP)) < 2.6 at Week 24 [Time Frame: Week 24]
Musculoskeletal	Change from Baseline in the Modified Total Sharp Score (mTSS) at Weeks 24 and 52 [Time Frame: Baseline; Weeks 24 and 52]
Musculoskeletal	Proportion of Participants who Achieve ACR 50% Improvement (ACR50) at Weeks 4, 12, 24, and 52 [Time Frame: Weeks 4, 12, 24, and 52]
Musculoskeletal	Proportion of Participants who Achieve ACR 70% Improvement (ACR70) at Weeks 4, 12, 24, and 52 [Time Frame: Weeks 4, 12, 24, and 52]
Musculoskeletal	Proportion of Participants who Achieve ACR20 at Weeks 4, 12, and 52 [Time Frame: Weeks 4, 12, and 52]
Musculoskeletal	Proportion of Participants who Achieve ACR20 Over Time from Day 1 through Week 52 [Time Frame: Up to 52 weeks]
Musculoskeletal	Proportion of Participants who Achieve ACR50 Over Time from Day 1 through Week 52 [Time Frame: Up to 52 weeks]
Musculoskeletal	Proportion of Participants who Achieve ACR70 Over Time from Day 1 through Week 52 [Time Frame: Up to 52 weeks]
Musculoskeletal	Change from Baseline in Individual Components of the ACR Response at Weeks 4, 12, 24, and 52 and Over Time from Day 1 through Week 52 [Time Frame: Baseline and up to 52 weeks]
Physical functioning	Proportion of Participants who Achieve Change in HAQ-DI of ≥ 0.22 at Weeks 4, 12, 24, and 52, and Over Time from Day 1 through Week 52 [Time Frame: Up to 52 weeks]
Musculoskeletal	Change from Baseline in DAS28 (CRP) at Weeks 4, 12, 24, and 52, and Over Time from Day 1 through Week 52 [Time Frame: Baseline and up to 52 weeks]
Musculoskeletal	Proportion of Participants who Achieve DAS28 (CRP) ≤ 3.2 at Weeks 4, 12, 24, and 52, and Over Time from Day 1 through Week 52 [Time Frame: Up to 52 weeks]
Musculoskeletal	Proportion of Participants who Achieve DAS28 (CRP) < 2.6 at Weeks 4, 12, and 52, and Over Time from Day 1 through Week 52 [Time Frame: Up to 52 weeks]
Musculoskeletal	American College of Rheumatology N (ACR-N) at Weeks 4, 12, 24, and 52, and Over Time from Day 1 through Week 52 [Time Frame: Up to 52 weeks]
Musculoskeletal	European League Against Rheumatism (EULAR) Response at Weeks 4, 12, 24, and 52, and Over Time from Day 1 through Week 52 [Time Frame: Up to 52 weeks]
Musculoskeletal	Change from Baseline in Clinical Disease Activity Index (CDAI) at Weeks 4, 12, 24, and 52, and Over Time from Day 1 through Week 52

Outcome classification	Outcomes
	[Time Frame: Baseline and up to 52 weeks]
Musculoskeletal	Change from Baseline in Simplified Diagnostic Activity Index (SDAI) at Weeks 4, 12, 24, and 52, and Over Time from Day 1 through Week 52 [Time Frame: Baseline and up to 52 weeks]
Musculoskeletal	Proportion of Participants with no Radiographic Progression from Baseline at Week 24 and 52 [Time Frame: Baseline; Weeks 24 and 52]
Physical functioning	Absolute Value and Change from Baseline in Short-form Health Survey (SF-36) at Weeks 4, 12, 24 and 52, and Over Time from Day 1 through Week 52 [Time Frame: Baseline and up to 52 weeks]
Social functioning	*Absolute Value and Change from Baseline in Short-form Health Survey (SF-36) at Weeks 4, 12, 24 and 52, and Over Time from Day 1 through Week 52 [Time Frame: Baseline and up to 52 weeks]*
Role functioning	*Absolute Value and Change from Baseline in Short-form Health Survey (SF-36) at Weeks 4, 12, 24 and 52, and Over Time from Day 1 through Week 52 [Time Frame: Baseline and up to 52 weeks]*
Emotional functioning/wellbeing	*Absolute Value and Change from Baseline in Short-form Health Survey (SF-36) at Weeks 4, 12, 24 and 52, and Over Time from Day 1 through Week 52 [Time Frame: Baseline and up to 52 weeks]*
Physical functioning	Absolute Value and Change from Baseline in the Functional Assessment of Chronic Illness Therapy-Fatigue Scale (FACIT-Fatigue) at Weeks 4, 12, 24 and 52, and Over Time from Day 1 through Week 52 [Time Frame: Baseline and up to 52 weeks]
Social functioning	*Absolute Value and Change from Baseline in the Functional Assessment of Chronic Illness Therapy-Fatigue Scale (FACIT-Fatigue) at Weeks 4, 12, 24 and 52, and Over Time from Day 1 through Week 52 [Time Frame: Baseline and up to 52 weeks]*
Physical functioning	Absolute Value and Change from Baseline in the EuroQol 5 Dimensions (EQ-5D) Patient-Reported Outcomes Survey at Weeks 4, 12, 24 and 52, and Over Time from Day 1 through Week 52 [Time Frame: Baseline and up to 52 weeks]
Emotional functioning/wellbeing	*Absolute Value and Change from Baseline in the EuroQol 5 Dimensions (EQ-5D) Patient-Reported Outcomes Survey at Weeks 4, 12, 24 and 52, and Over Time from Day 1 through Week 52 [Time Frame: Baseline and up to 52 weeks]*
Physical functioning	Absolute Value and Change from Baseline in Work Productivity and Activity Impairment - Rheumatoid Arthritis (WPAI-RA) Patient-Reported Outcomes Survey at Weeks 4, 12, 24, and 52, and Over Time from Day 1 through Week 52 [Time Frame: Baseline and up to 52 weeks]
Role functioning	*Absolute Value and Change from Baseline in Work Productivity and Activity Impairment - Rheumatoid Arthritis (WPAI-RA) Patient-Reported Outcomes Survey at Weeks 4, 12, 24, and 52, and Over Time from Day 1 through Week 52 [Time Frame: Baseline and up to 52 weeks]*

Study 3: Filgotinib in Combination With Methotrexate in Adults With Moderately to Severely Active Rheumatoid Arthritis Who Have an Inadequate Response to Methotrexate

Outcome classification	Outcomes
Musculoskeletal	Proportion of Participants who Achieve an American College of Rheumatology (ACR) 20% Improvement (ACR20) Response at Week 12 [Time Frame: Week 12]
Musculoskeletal	Proportion of Participants who Achieve Disease Activity Score based on 28 joints (DAS28) (C-reactive protein (CRP)) ≤ 3.2 at Week 12 [Time Frame: Week 12]
Physical functioning	Change from Baseline in the Health Assessment Questionnaire - Disability Index (HAQ-DI) Score at Week 12 [Time Frame: Week 12]
Musculoskeletal	Proportion of Participants who Achieve DAS28 (CRP) < 2.6 at Week 24 [Time Frame: Week 24]
Musculoskeletal	Change from Baseline in the Modified Total Sharp Score (mTSS) at Week 24 [Time Frame: Week 24]
Musculoskeletal	Proportion of Participants who Achieve ACR 50% Improvement (ACR50) at Weeks 4, 12, 24, and 52 [Time Frame: Weeks 4, 12, 24, and 52]
Musculoskeletal	Proportion of Participants who Achieve ACR 70% Improvement (ACR70) at Weeks 4, 12, 24, and 52 [Time Frame: Weeks 4, 12, 24, and 52]
Musculoskeletal	Proportion of Participants who Achieve ACR20 at Weeks 4, 24, and 52 [Time Frame: Weeks 4, 24, and 52]
Musculoskeletal	Proportion of Participants who Achieve ACR20 Over Time from Day 1 through Week 52 [Time Frame: Up to 52 weeks]
Musculoskeletal	Proportion of Participants who Achieve ACR50 Over Time from Day 1 through Week 52 [Time Frame: Up to 52 weeks]
Musculoskeletal	Proportion of Participants who Achieve ACR70 Over Time from Day 1 through Week 52 [Time Frame: Up to 52 weeks]
Musculoskeletal	Change from Baseline in Individual Components of the ACR Response at Weeks 4, 12, 24, and 52 and Over Time from Day 1 through Week 52 [Time Frame: Baseline and up to 52 weeks]
Physical functioning	Proportion of Participants who Achieve Change in HAQ-DI of ≥ 0.22 at Weeks 4, 12, 24, and 52, and Over Time from Day 1 through Week 52 [Time Frame: Up to 52 weeks]
Musculoskeletal	Change from Baseline in DAS28 (CRP) at Weeks 4, 12, 24, and 52, and Over Time from Day 1 through Week 52 [Time Frame: Baseline and up to 52 weeks]
Musculoskeletal	Proportion of Participants who Achieve DAS28 (CRP) ≤ 3.2 at Weeks 4, 24, and 52, and Over Time from Day 1 through Week 52 [Time Frame: Up to 52 weeks]
Musculoskeletal	Proportion of Participants who Achieve DAS28 (CRP) < 2.6 at Weeks 4, 12, and 52, and over time from Day 1 through Week 52 [Time Frame: Up to 52 weeks]
Musculoskeletal	American College of Rheumatology N (ACR-N) at Weeks 4, 12, 24, and 52, and over time from Day 1 through Week 52 [Time Frame: Up to 52 weeks]
Musculoskeletal	European League Against Rheumatism (EULAR) Response at Weeks 4, 12, 24, and 52, and over time from Day 1 through Week 52 [Time Frame: Up to 52 weeks]
Musculoskeletal	Change from Baseline in Clinical Disease Activity Index (CDAI) at Weeks 4, 12, 24, and 52, and Over Time from Day 1 through Week 52 [Time Frame: Baseline and up to 52 weeks]

**Outcome classification**	**Outcomes**
Musculoskeletal	Change from Baseline in Simplified Diagnostic Activity Index (SDAI) at Weeks 4, 12, 24, and 52, and Over Time from Day 1 through Week 24 [Time Frame: Baseline and up to 24 weeks]
Musculoskeletal	Change from Baseline in the mTSS at Week 52 [Time Frame: Baseline; Week 52]
Musculoskeletal	Proportion of Participants with No Radiographic Progression from Baseline at Weeks 24 and 52 [Time Frame: Baseline; Weeks 24 and 52]
Physical functioning	Absolute Value and Change from Baseline in Short-form Health Survey (SF-36) at Weeks 4, 12, 24, and 52, and Over Time from Day 1 through Week 52 [Time Frame: Baseline and up to 52 weeks]
Social functioning	*Absolute Value and Change from Baseline in Short-form Health Survey (SF-36) at Weeks 4, 12, 24, and 52, and Over Time from Day 1 through Week 52 [Time Frame: Baseline and up to 52 weeks]*
Role functioning	*Absolute Value and Change from Baseline in Short-form Health Survey (SF-36) at Weeks 4, 12, 24, and 52, and Over Time from Day 1 through Week 52 [Time Frame: Baseline and up to 52 weeks]*
Emotional functioning/wellbeing	*Absolute Value and Change from Baseline in Short-form Health Survey (SF-36) at Weeks 4, 12, 24, and 52, and Over Time from Day 1 through Week 52 [Time Frame: Baseline and up to 52 weeks]*
Physical functioning	Absolute Value and Change from Baseline in the Functional Assessment of Chronic Illness Therapy-Fatigue Scale (FACIT-Fatigue) at Weeks 4, 12, 24, and 52, and Over Time from Day 1 through Week 52 [Time Frame: Baseline and up to 52 weeks]
Social functioning	*Absolute Value and Change from Baseline in the Functional Assessment of Chronic Illness Therapy-Fatigue Scale (FACIT-Fatigue) at Weeks 4, 12, 24, and 52, and Over Time from Day 1 through Week 52 [Time Frame: Baseline and up to 52 weeks]*
Physical functioning	Absolute Value and Change from Baseline in the EuroQol 5 Dimensions (EQ-5D) Patient-Reported Outcomes Survey at Weeks 4, 12, 24, and 52, and Over Time from Day 1 through Week 52 [Time Frame: Baseline and up to 52 weeks]
Emotional functioning/wellbeing	*Absolute Value and Change from Baseline in the EuroQol 5 Dimensions (EQ-5D) Patient-Reported Outcomes Survey at Weeks 4, 12, 24, and 52, and Over Time from Day 1 through Week 52 [Time Frame: Baseline and up to 52 weeks]*
Physical functioning	Absolute Value and Change from Baseline in Work Productivity and Activity Impairment - Rheumatoid Arthritis (WPAI-RA) Patient-Reported Outcomes Survey at Weeks 4, 12, 24, and 52, and Over Time from Day 1 through Week 52 [Time Frame: Baseline and up to 52 weeks]
Role functioning	*Absolute Value and Change from Baseline in Work Productivity and Activity Impairment - Rheumatoid Arthritis (WPAI-RA) Patient-Reported Outcomes Survey at Weeks 4, 12, 24, and 52, and Over Time from Day 1 through Week 52 [Time Frame: Baseline and up to 52 weeks]*

Study 4: Filgotinib Versus Placebo in Adults With Active Rheumatoid Arthritis (RA) Who Have an Inadequate Response to Biologic Disease-modifying Anti-rheumatic Drug(s) (DMARDs) Treatment

Outcome classification	Outcomes
Musculoskeletal	Proportion of Participants who Achieve an American College of Rheumatology (ACR) 20% Improvement (ACR20) Response at Week 12 [Time Frame: Week 12]
Musculoskeletal	Proportion of Participants who Achieve Disease Activity Score based on 28 joints (DAS28) (C-reactive protein (CRP)) ≤ 3.2 at Week 12 [Time Frame: Week 12]
Physical functioning	Change from Baseline in the Health Assessment Questionnaire - Disability Index (HAQ-DI) Score at Week 12 [Time Frame: Week 12]
Musculoskeletal	Proportion of Participants who Achieve ACR 50% Improvement (ACR50) at Weeks 4, 12, and 24 [Time Frame: Weeks 4, 12, and 24]
Musculoskeletal	Proportion of Participants who Achieve ACR 70% Improvement (ACR70) at Weeks 4, 12, and 24 [Time Frame: Weeks 4, 12, and 24]
Musculoskeletal	Proportion of Participants who Achieve ACR20 at Weeks 4 and 24 [Time Frame: Weeks 4 and 24]
Musculoskeletal	Proportion of Participants who Achieve ACR20 Over Time from Day 1 through Week 24 [Time Frame: Up to 24 weeks]
Musculoskeletal	Proportion of Participants who Achieve ACR50 Over Time from Day 1 through Week 24 [Time Frame: Up to 24 weeks]
Musculoskeletal	Proportion of Participants who Achieve ACR70 Over Time from Day 1 through Week 24 [Time Frame: Up to 24 weeks]
Musculoskeletal	Change from Baseline in Individual Components of the ACR Response at Weeks 4, 12, and 24 and Over Time from Day 1 through Week 24 [Time Frame: Baseline and up to 24 weeks]
Physical functioning	Proportion of Participants who Achieve Change in HAQ-DI of ≥ 0.22 at Weeks 4, 12, and 24, and Over Time from Day 1 through Week 24 [Time Frame: Up to 24 weeks]
Musculoskeletal	Change from Baseline in DAS28 (CRP) at Weeks 4, 12, and 24, and Over Time from Day 1 through Week 24 [Time Frame: Baseline and up to 24 weeks]
Musculoskeletal	Proportion of Participants who Achieve DAS28 (CRP) ≤ 3.2 at Weeks 4, and 24, and Over Time from Day 1 through Week 24 [Time Frame: Up to 24 weeks]
Musculoskeletal	Proportion of Participants who Achieve DAS28 (CRP) < 2.6 at Weeks 4, and 24, and Over Time from Day 1 through Week 24 [Time Frame: Up to 24 weeks]
Musculoskeletal	American College of Rheumatology N (ACR-N) at Weeks 4, 12, and 24, and Over Time from Day 1 through Week 24 [Time Frame: Up to 24 weeks]
Musculoskeletal	European League Against Rheumatism (EULAR) Response at Weeks 4, 12, and 24, and Over Time from Day 1 through Week 24 [Time Frame: Up to 24 weeks]
Musculoskeletal	Change from Baseline in Clinical Diagnostic Activity Index (CDAI) at Weeks 4, 12, and 24, and Over Time from Day 1 through Week 24 [Time Frame: Baseline and up to 24 weeks]
Musculoskeletal	Change from Baseline in Simplified Diagnostic Activity Index (SDAI) at Weeks 4, 12, and 24, and over time from Day 1 through Week 24 [Time Frame: Baseline and up to 24 weeks]
Physical functioning	Absolute Value and Change from Baseline in Short-form Health Survey (SF-36) at Weeks 4, 12 and 24, and Over Time from Day 1 through Week

Outcome classification	Outcomes
	24 [Time Frame: Baseline and up to 24 weeks]
Social functioning	*Absolute Value and Change from Baseline in Short-form Health Survey (SF-36) at Weeks 4, 12 and 24, and Over Time from Day 1 through Week 24 [Time Frame: Baseline and up to 24 weeks]*
Role functioning	*Absolute Value and Change from Baseline in Short-form Health Survey (SF-36) at Weeks 4, 12 and 24, and Over Time from Day 1 through Week 24 [Time Frame: Baseline and up to 24 weeks]*
Emotional functioning/wellbeing	*Absolute Value and Change from Baseline in Short-form Health Survey (SF-36) at Weeks 4, 12 and 24, and Over Time from Day 1 through Week 24 [Time Frame: Baseline and up to 24 weeks]*
Physical functioning	Absolute Value and Change from Baseline in the Functional Assessment of Chronic Illness Therapy-Fatigue Scale (FACIT-Fatigue) at Weeks 4, 12 and 24, and Over Time from Day 1 through Week 24 [Time Frame: Baseline and up to 24 weeks]
Social functioning	*Absolute Value and Change from Baseline in the Functional Assessment of Chronic Illness Therapy-Fatigue Scale (FACIT-Fatigue) at Weeks 4, 12 and 24, and Over Time from Day 1 through Week 24 [Time Frame: Baseline and up to 24 weeks]*
Physical functioning	Absolute Value and Change from Baseline in the EuroQol 5 Dimensions (EQ-5D) Patient-Reported Outcomes Survey at Weeks 4, 12 and 24, and Over Time from Day 1 through Week 24 [Time Frame: Baseline and up to 24 weeks]
Emotional functioning/wellbeing	*Absolute Value and Change from Baseline in the EuroQol 5 Dimensions (EQ-5D) Patient-Reported Outcomes Survey at Weeks 4, 12 and 24, and Over Time from Day 1 through Week 24 [Time Frame: Baseline and up to 24 weeks]*
Physical functioning	Absolute Value and Change from Baseline in Work Productivity and Activity Impairment- Rheumatoid Arthritis (WPAI-RA) at Weeks 4, 12, 24, and Over Time from Day 1 through Week 24 [Time Frame: Baseline and up to 24 weeks]
Role functioning	*Absolute Value and Change from Baseline in Work Productivity and Activity Impairment- Rheumatoid Arthritis (WPAI-RA) at Weeks 4, 12, 24, and Over Time from Day 1 through Week 24 [Time Frame: Baseline and up to 24 weeks]*

Study 5: Comparison of Disease Modifying Antirheumatic Drugs Therapy in Patients With RA Failing Methotrexate Monotherapy

Outcome classification	Outcomes
Musculoskeletal	Good response according to European league against rheumatism (EULAR) response [Time Frame: 3 months]
Musculoskeletal	Disease activity as per Ultrasound-7 (US-7) score [Time Frame: 3 months]
Musculoskeletal	Radiographic damage by SENS scoring system [Time Frame: 3 months]
Adverse events	Adverse drug reactions [Time Frame: 3 months]
Physical functioning	Indian health assessment questionnaire (iHAQ) [Time Frame: 3 months]

Study 6: Study to Assess if ABP710 is Safe & Effective in Treating Moderate to Severe Rheumatoid Arthritis Compared to Infliximab

Outcome classification	Outcomes
Musculoskeletal	Response difference measured by 20% improvement in ACR core set measurements (ACR20) (Various Time Frames]
Musculoskeletal	Response difference measured by 50% improvement in ACR core set measurements (ACR50) [Various Time Frames]
Musculoskeletal	Response difference measured by 70% improvement in ACR core set measurements (ACR70) [Various Time Frames]
Musculoskeletal	Change in disease activity measured by the disease activity score in 28 joints - C-reactive protein (DAS28-CRP) [Various Time Frames]
Adverse events	Treatment-emergent adverse events, serious adverse events, and adverse events of special interest [Time Frame: Baseline through Week 50]
Musculoskeletal	Clinically significant changes in laboratory values and vital signs [Time Frame: Baseline through Week 50]
Immune system	Incidence of antidrug antibodies at baseline [Various Time Frames]
Musculoskeletal	Trough serum concentrations of ABP 710 and infliximab at week 2 [Various Time Frames]

Study 7: A Multicenter, 2 Part Study to Assess the Efficacy and Safety of H.P. Acthar® Gel in Subjects With Rheumatoid Arthritis

Outcome classification	Outcomes
Musculoskeletal	Proportion of subjects with Disease Activity Score with 28 joint count and Erythrocyte Sedimentation Rate (DAS28-ESR) [Time Frame: 12 Weeks]
Musculoskeletal	Proportion of subjects with DAS28-ESR <3.2 at Week 12
Musculoskeletal	Proportion of subjects who maintained DAS28-ESR [Time Frame: 24 Weeks]
Musculoskeletal	Proportion of subjects with Low Disease Activity as defined by DAS28-ESR <3.2 at Week 24

Study 8: Assess the Injection Site Pain Associated With a New Etanercept Formulation in Adult Subjects With RA or PsA

Outcome classification	Outcomes
General disorders	Number of participants with injection site pain as assessed by visual analog scale [Time Frame: Through week 2]
General disorders	Number of participants with injection site pain as assessed by visual analog scale per disease indication [Time Frame: Through week 2]
Adverse events	Number of participants with adverse events [Time Frame: Through day 38]

Study 9: A Study of the Efficacy and Safety of TACI-antibody Fusion Protein Injection (RC18) in Subjects With Inadequate Response to MTX Due to Treat Moderate and Severe Rheumatoid Arthritis

Outcome classification	Outcomes
Musculoskeletal	The proportion of patients in each group reached ACR20 24 weeks for visits [Time Frame: Week 24（Visit 9）]
Musculoskeletal	Percentage of Participants Achieving American College of Rheumatology ACR50 and ACR70 Responses at week 24. [Time Frame: Week 24]
Musculoskeletal	Percentage of Participants Achieving Low Disease Activity and clinical remission. (DAS28 ≤3.20 and DAS28 < 2.6). [Time Frame: Week 24]
Musculoskeletal	Percentage of Participants Achieving American College of Rheumatology ACR50 and ACR70 Responses at week 12 or week 24. [Time Frame: Week 12 and Week 24]
Musculoskeletal	Sharp Score Relative Change from Baseline at Week 24 [Time Frame: Week 24]
Musculoskeletal	Percentage of Participants With American College of Rheumatology 20% ,50% and 70% (ACR20, ACR50 and ACR70) Response [Time Frame: Week 4, Week 8, Week 12, Week 28, Week 32, Week 40, Week 48]
Musculoskeletal	Change From Baseline in Joint Space Narrowing and Erosions at week 24 and week 48. [Time Frame: Week 24, Week 48]

Study 10: A Study to Assess the Efficacy and Safety of Abatacept in Adults With Active Primary Sjögrens Syndrome

Outcome classification	Outcomes
Musculoskeletal	Change from baseline in EULAR Sjögren's Syndrome Disease Activity Index (ESSDAI) [Time Frame: Day 1 to Day 169]
Musculoskeletal	Change from baseline in EULAR Sjögren's Syndrome Patient Reported Index (ESSPRI) [Time Frame: Day 1 to Day 169]
Musculoskeletal	Mean change from baseline in the stimulated whole salivary flow among subjects with stimulated whole salivary flow of at least 0.1 mL/min at both screening and baseline [Time Frame: Day 1 to Day 169]
Immune system	Proportion of subjects with a least one positive immunogenicity response as measured by anti-abatacept antibody testing [Time Frame: Up to 1 year]
Adverse events	Proportion of subjects with adverse events (AEs) [Time Frame: Day 1 to 56 days after the last dose of study drug]
Adverse events	Proportion of subjects with serious adverse events (SAEs) [Time Frame: Day 1 to 56 days after the last dose of study drug]
Delivery of care	Proportion of subjects with AEs leading to discontinuation [Time Frame: Day 1 to 56 days after the last dose of study drug]
Mortality/survival	Proportion of Deaths [Time Frame: Day 1 to 56 days after the last dose of study drug]
Musculoskeletal	Proportion of subjects with laboratory abnormalities [Time Frame: Day 1 to 56 days after the last dose of study drug]
